# Supplementary material for: Boosting anti-PD-1 therapy with metformin-loaded macrophage-derived microparticles
Source: Nat Commun. 2021 Jan 19;12:440. doi: 10.1038/s41467-020-20723-x (PMC7815730; doi:10.1038/s41467-020-20723-x)
Supplement: Supplementary file 1 — Supplementary Information [file 41467_2020_20723_MOESM1_ESM.pdf]

Supplementary Information

Boosting Anti-PD-1 Therapy with Metformin-Loaded Macrophage-Derived Microparticles

Zhaohan Wei[1,8], Xiaoqiong Zhang[1,8], Tuying Yong[1,2,3], Nana Bie[1], Guiting Zhan[1], Xin Li[1], Qingle Liang[1], Jianye Li[1], Jingjing Yu[4], Gang Huang[5], Yuchen Yan[1], Zelong Zhang[1], Bixiang Zhang[4], Lu Gan[1,2,3]*, Bo Huang[6,7]*, Xiangliang Yang[1,2,3]*

[1]National Engineering Research Center for Nanomedicine, College of Life Science and Technology, Huazhong University of Science and Technology, Wuhan 430074, China

[2]Key Laboratory of Molecular Biophysics of the Ministry of Education, College of Life Science and Technology, Huazhong University of Science and Technology, Wuhan 430074, China

[3]Hubei Key Laboratory of Bioinorganic Chemistry and Materia Medica, Huazhong University of Science and Technology, Wuhan 430074, China

[4]Hepatic Surgery Center, Tongji Hospital, Tongji Medical College, Huazhong University of Science and Technology, Wuhan 430030, China

[5]School Hospital, Huazhong University of Science and Technology, Wuhan 430074, China

[6]Department of Immunology & National Key Laboratory of Medical Molecular Biology, Institute of Basic Medical Sciences, Chinese Academy of Medical Sciences, Peking Union Medical College, Beijing 100005, China

[7]Department of Biochemistry and Molecular Biology, Tongji Medical College, Huazhong University of Science and Technology, Wuhan 430030, China

[8]Z. Wei and X. Zhang contributed equally to this work.

*Correspondence should be addressed to: lugan@mail.hust.edu.cn, tjhuangbo@hotmail.com, yangxl@mail.hust.edu.cn

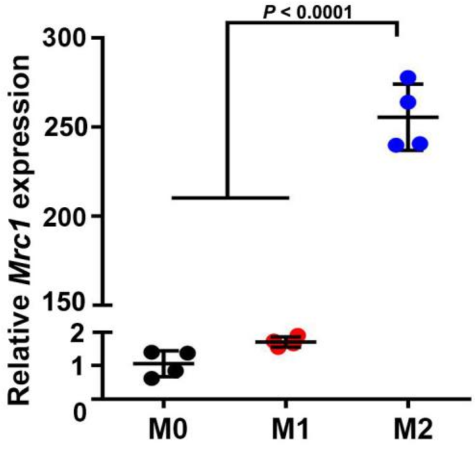

Supplementary Figure 1. Overexpression of *Mrc1* in M2-like macrophages.

Mrc1 (mannose receptor C-type 1) mRNA expression levels in RAW264.7 cells (M0 macrophages), RAW264.7 cells stimulated with 100 ng mL^{-1} LPS and 20 ng mL^{-1} IFN-γ for 24 h (M1-like macrophages), and RAW264.7 cells stimulated with 20 ng mL^{-1} IL-4 for 24 h (M2-like macrophages) by real time RT-PCR analysis. Data are presented as means ± s.d. (n=4 biologically independent samples; one-way ANOVA followed by Tukey's HSD post-hoc test). Source data are provided as a Source Data file.

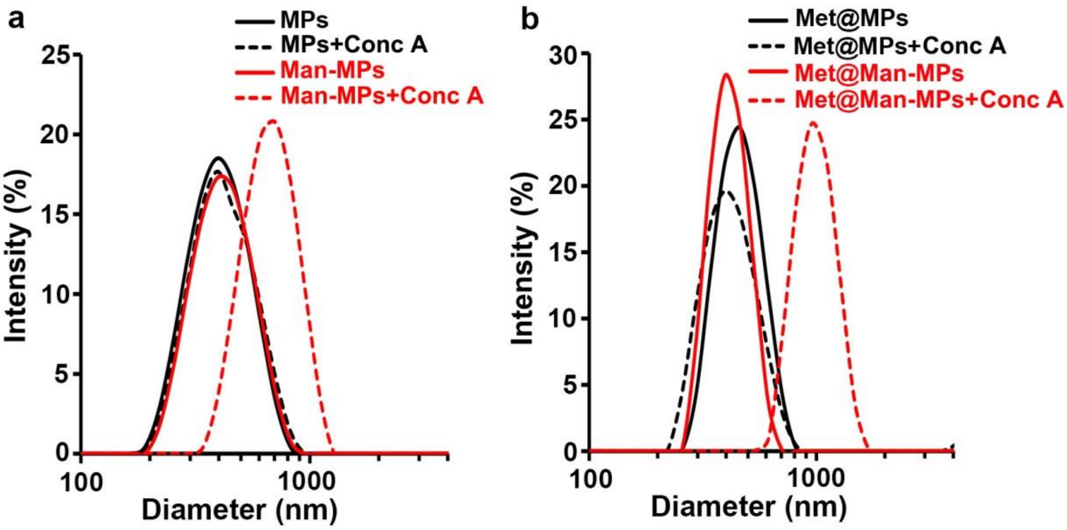

Supplementary Figure 2. Confirmation of mannose modification in Man-MPs and Met@Man-MPs.

(**a**) Diameters of MPs and Man-MPs after incubation with or without Concanavalin A (Conc A, 1 mg mL^{-1}) for 2 h by DLS analysis. (**b**) Diameters of Met@MPs and Met@Man-MPs after incubation with or without Conc A (1 mg mL^{-1}) for 2 h by DLS analysis. Source data are provided as a Source Data file.

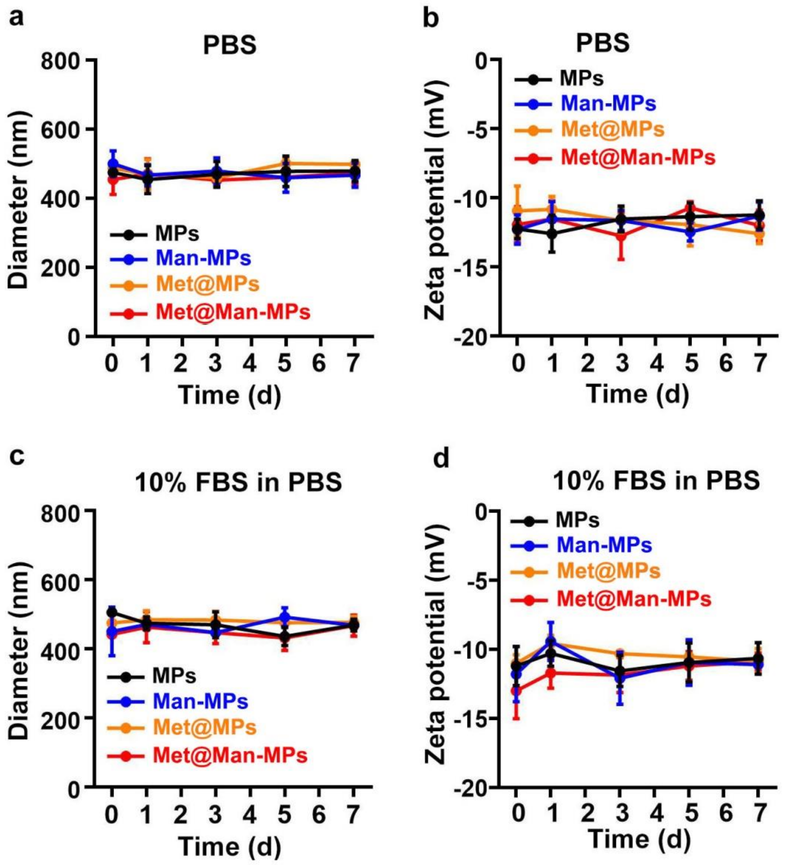

Supplementary Figure 3. Stability of Met@Man-MPs.

(**a,b**) Diameter (**a**) and zeta potential (**b**) of MPs and Man-MPs with or without Met after incubation in PBS for different time intervals. Data are presented as mean ± s.d. (n=3 independent experiments). (**c,d**) Diameter (**c**) and zeta potential (**d**) of MPs and Man-MPs with or without Met after incubation in PBS containing 10% FBS for different time intervals. Data are presented as mean ± s.d. (n=3 independent experiments). Source data are provided as a Source Data file.

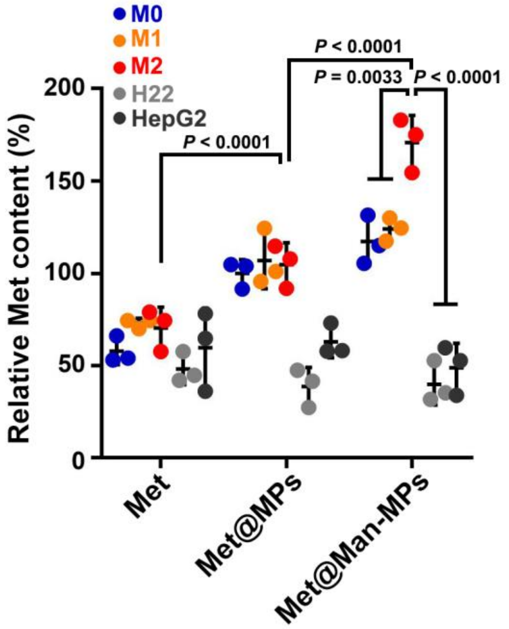

Supplementary Figure 4. M2 macrophage targeting capacity of Met@Man-MPs.

Relative Met content in RAW264.7 cells (M0 macrophages), LPS- and IFN-γ-conditioned RAW264.7 cells (M1-like macrophages), IL-4-conditioned RAW264.7 cells (M2-like macrophages), H22 and HepG2 cells after treatment with free Met, Met@MPs or Met@Man-MPs at Met concentration of 40 μg mL^{-1} for 4 h by HPLC. Data are presented as means ± s.d. (n=3 biologically independent samples; two-way ANOVA followed by Bonferroni's multiple comparisons post-test). Source data are provided as a Source Data file.

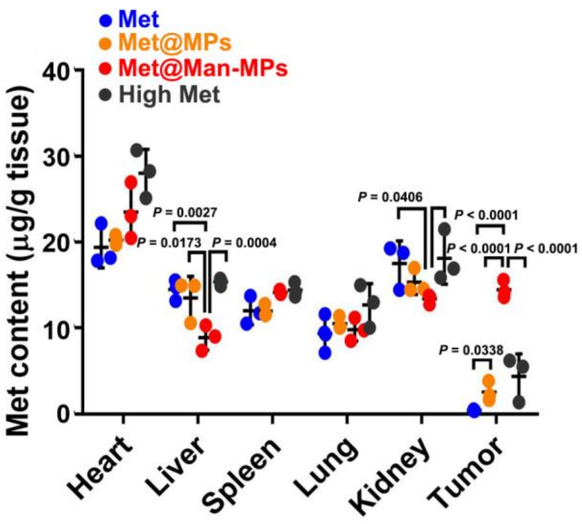

Supplementary Figure 5. Tumor targeting of Met@Man-MPs.

Met concentration in different organs and tumors of H22 tumor-bearing mice at 24 h after intravenous injection of free Met, Met@MPs or Met@Man-MPs at the Met dosage of 10 mg kg^{-1}, or high dosage of free Met at 100 mg kg^{-1} by HPLC. Data are presented as means ± s.d. (n=3 mice per group; one-way ANOVA followed by Tukey's HSD post-hoc test). Source data are provided as a Source Data file.

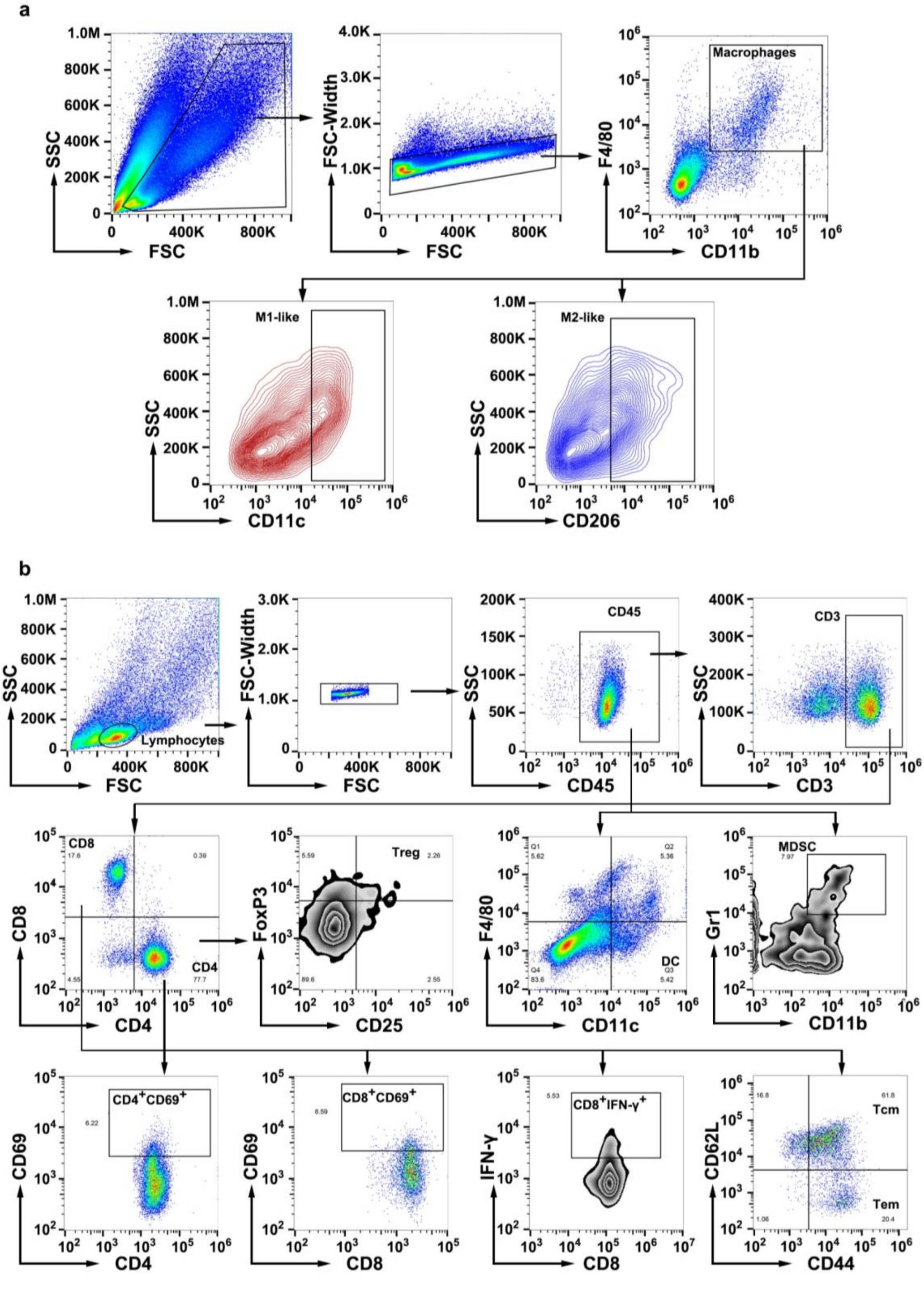

Supplementary Figure 6. Gating strategy for identifying major immune cell populations in tumor tissues of tumor-bearing mice.

(**a**) Gating strategy for identifying M1- and M2-like TAMs in tumor tissues of tumor-bearing mice presented on Fig. 2f, 4d, 4e, 9c, 9d and Supplementary Fig. 26a,

26b, 27a. (**b**) Gating strategy for identifying $CD4^+$ T cells, $CD4^+CD69^+$ T cells, $CD8^+$ T cells, $CD8^+CD69^+$ T cells, $CD8^+$IFN-γ^+ T cells, DCs, Tregs, MDSCs and $CD8^+$ effector memory T cells in tumor tissues of tumor-bearing mice presented on Fig. 2f, 4f-k, 6d-f, 8j, 9e-j and Supplementary Fig. 26c-h, 27c-g.

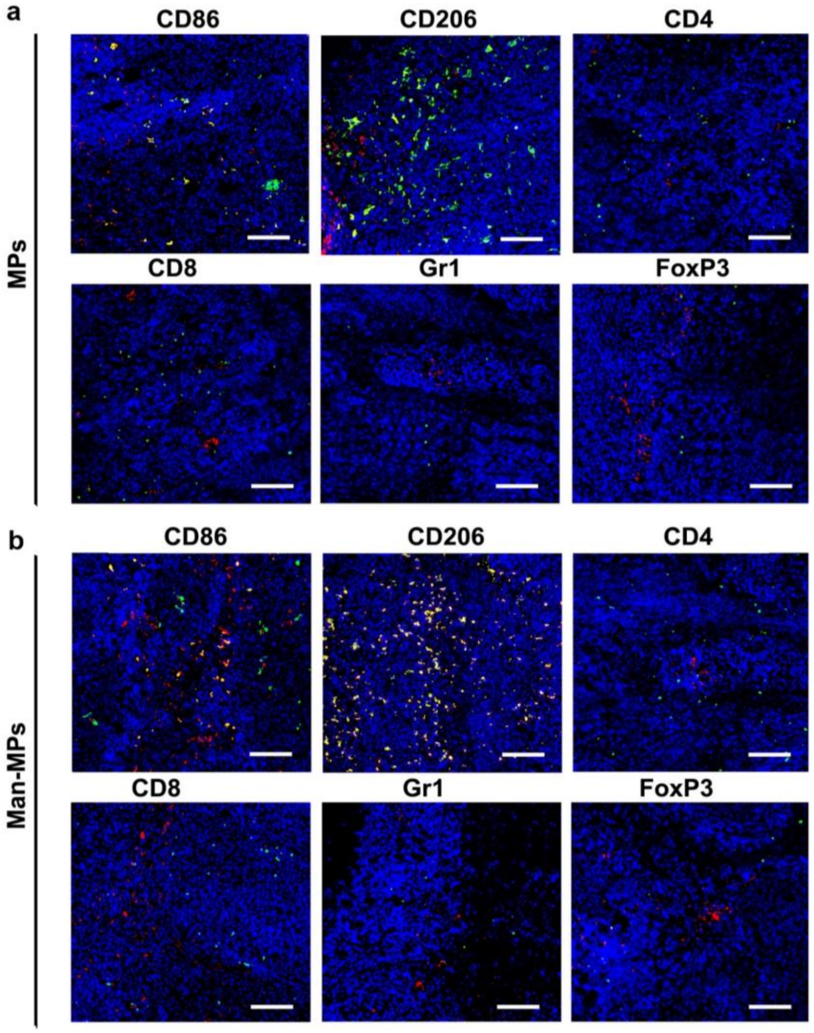

Supplementary Figure 7. M2-like TAM targeting of Met@Man-MPs.

(**a,b**) Colocalization of PKH26-labelled MPs (**a**) or Man-MPs (**b**, red) with M1-like TAMs (labelling with Cy5-conjugated CD86 antibody, green pseudo color), M2-like TAMs (labelling with FITC-conjugated CD206 antibody, green), $CD4^{+}$ T cells (labelling with FITC-conjugated CD4 antibody, green), $CD8^{+}$ T cells (labelling with Cy5-conjugated CD8 antibody, green pseudo color), MDSCs (labelling with Cy5-conjugated Gr1 antibody, green pseudo color) and Tregs (labelling with FITC-conjugated FoxP3 antibody, green) in tumor tissues of H22 tumor-bearing mice at 24 h after intravenous injection of PKH26-labelled MPs or Man-MPs at the dosage of 15 mg protein kg^{-1}. The nuclei were stained with DAPI (blue). Images are representative of 3 independent experiments. Scale bars: 100 μm.

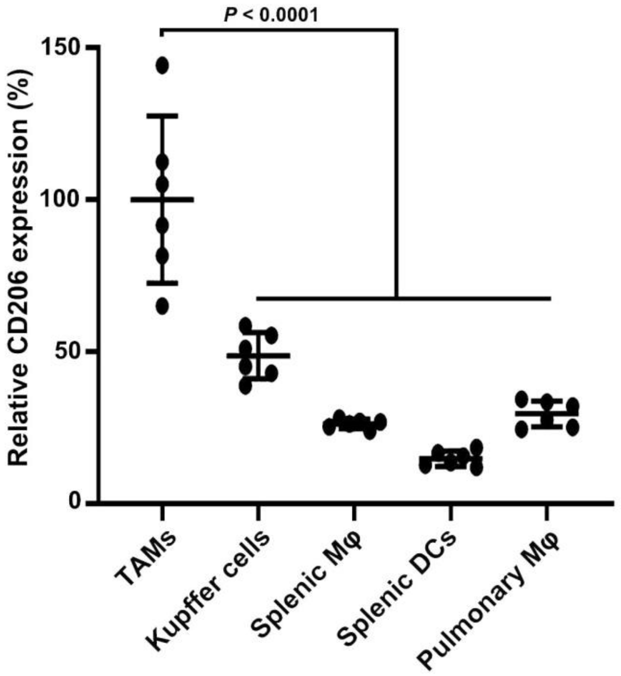

Supplementary Figure 8. CD206 expression in tissue-resident macrophages and phagocytes.

CD206 expression in TAMs, liver Kupffer cells, splenic macrophages, pulmonary macrophages (all these macrophages including TAMs were gated as $CD11b^{+}F4/80^{+}$ cells) and splenic DCs ($CD45^{+}F4/80^{-}CD11c^{+}$ cells) of H22 tumor-bearing mice by flow cytometry. Data are presented as means ± s.d. (n=6 mice per group; one-way ANOVA followed by Tukey's HSD post-hoc test). Source data are provided as a Source Data file.

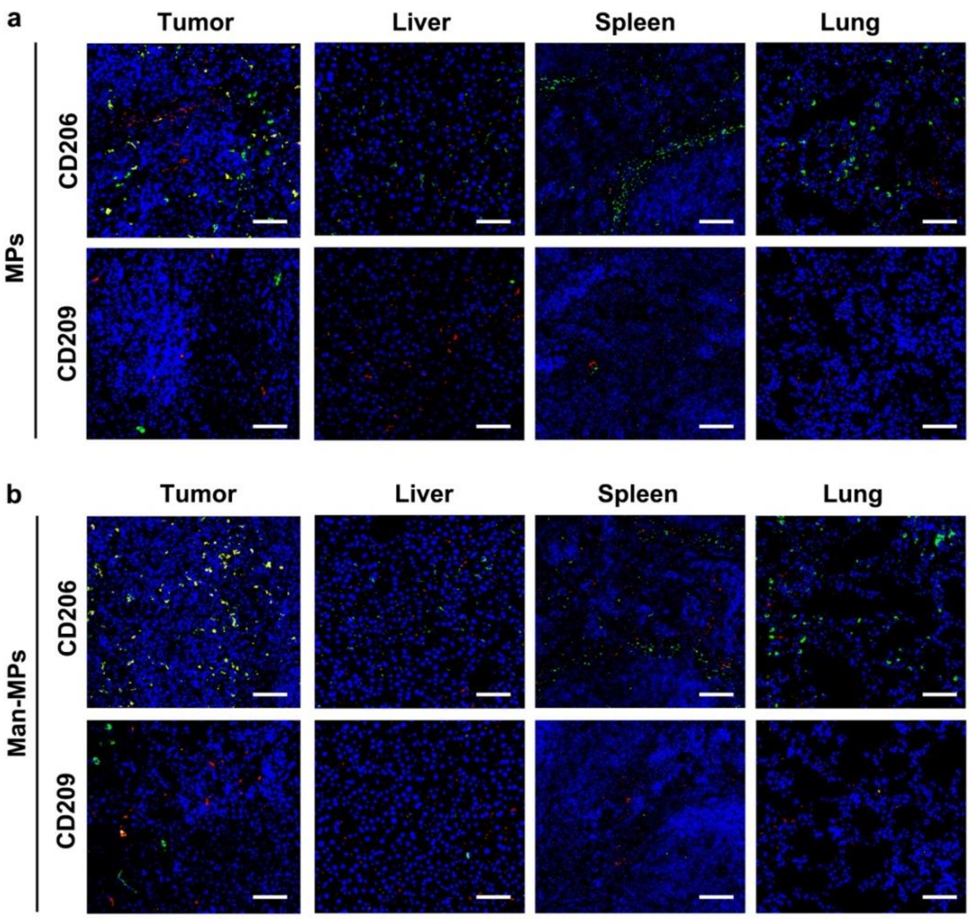

Supplementary Figure 9. CD206^{+} cell targeting of Man-MPs in tumors.

(**a,b**) Colocalization of PKH26-labelled MPs (**a**) or Man-MPs (**b**, red) with CD206- or CD209-positive cells labelled with Cy3-conjugated CD206 or CD209 antibody (green) in tumors, livers, spleens or lungs of H22 tumor-bearing mice at 24 h after intravenous injection of PKH26-labelled MPs or Man-MPs at the dosage of 15 mg protein kg^{-1}. The nuclei were stained with DAPI (blue). Images are representative of 3 independent experiments. Scale bars: 100 μm.

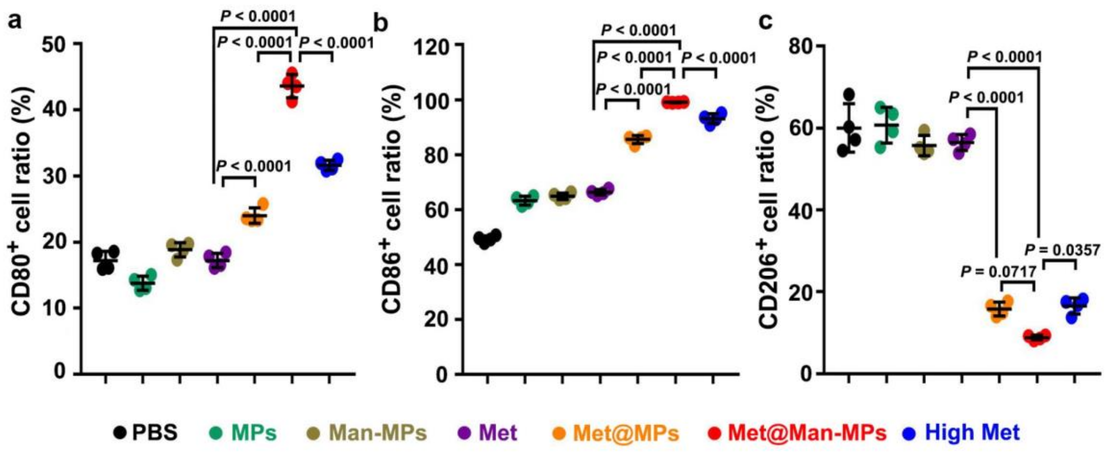

Supplementary Figure 10. Repolarization of M2-like macrophages to M1 phenotype by Met@Man-MPs in IL-4-conditioned RAW264.7 cells.

(**a**-**c**) Protein expression of CD80 (**a**), CD86 (**b**) and CD206 (**c**) in IL-4-conditioned RAW264.7 cells after treatment with PBS, MPs, Man-MPs, free Met, Met@MPs or Met@Man-MPs at the Met concentration of 20 μg mL^{-1}, or high concentration of Met at 200 μg mL^{-1} for 24 h by flow cytometry. Data are presented as means ± s.d. (n=4 biologically independent samples; one-way ANOVA followed by Tukey's HSD post-hoc test). Source data are provided as a Source Data file.

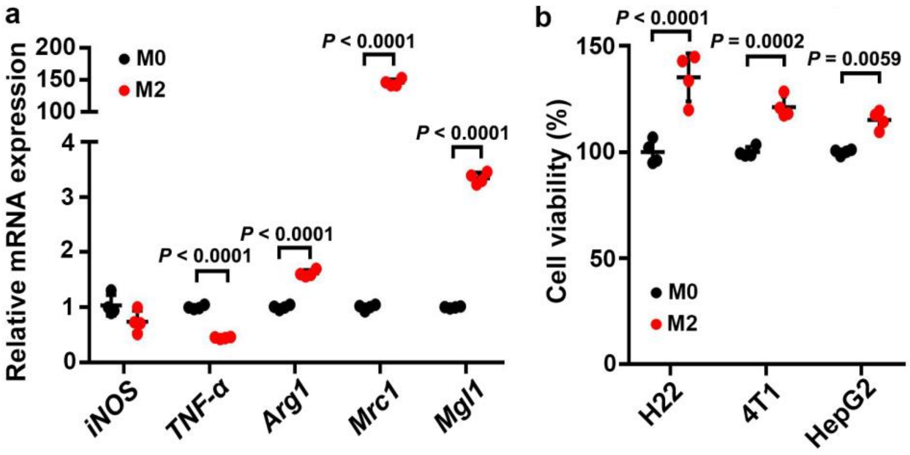

Supplementary Figure 11. Characterization of IL-4-conditioned RAW264.7 cells.

(**a**) The mRNA expression of M1- and M2-related markers in RAW264.7 cells (M0 macrophages) and IL-4-conditioned RAW264.7 cells (M2-like macrophages). Data are presented as means ± s.d. (n=4 biologically independent samples; unpaired two-tailed Student's *t*-test). (**b**) Cell viability of H22, 4T1 and HepG2 cells after treatment with the supernatants of RAW264.7 cells or IL-4-conditioned RAW264.7 cells for 24 h. Data are presented as means ± s.d. (n=4 biologically independent samples; unpaired two-tailed Student's *t*-test). Source data are provided as a Source Data file.

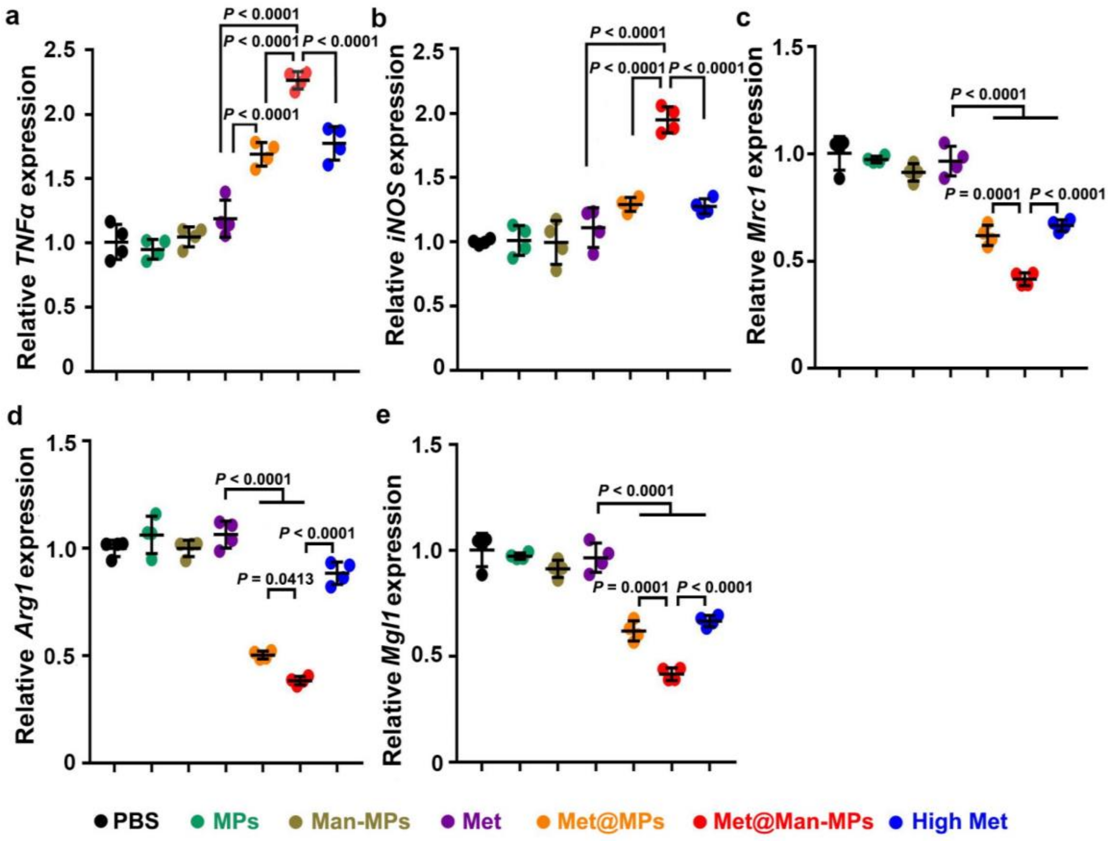

Supplementary Figure 12. Repolarization of M2-like macrophages to M1 phenotype by Met@Man-MPs derived from RAW264.7 cells in IL-4-conditioned BMDMs.

(**a-e**) mRNA expression levels of *TNFα* (**a**), *iNOS* (**b**), *Mrc1* (**c**), *Arg1* (**d**) and *Mgl1* (**e**) in IL-4-conditioned BMDMs after treatment with PBS, MPs, Man-MPs, free Met, Met@MPs or Met@Man-MPs derived from RAW264.7 cells at the Met concentration of 20 μg mL^{-1}, or high concentration of Met at 200 μg mL^{-1} for 24 h by real time RT-PCR. Data are presented as means ± s.d. (n=4 biologically independent samples; one-way ANOVA followed by Tukey's HSD post-hoc test). Source data are provided as a Source Data file.

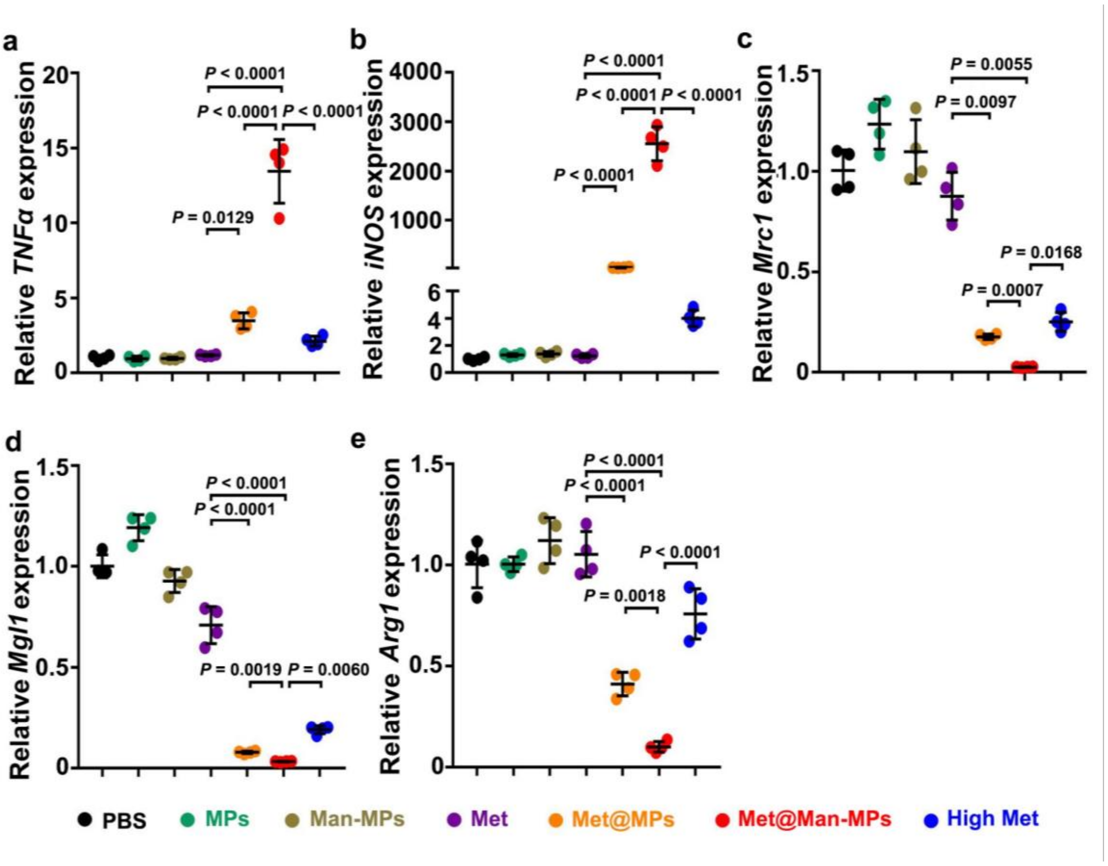

Supplementary Figure 13. Repolarization of M2-like macrophages to M1 phenotype by Met@Man-MPs derived from BMDMs in IL-4-conditioned BMDMs.

(**a-e**) mRNA expression levels of *TNFα* (**a**), *iNOS* (**b**), *Mrc1* (**c**), *Mgl1* (**d**) and *Arg1* (**e**) in IL-4-conditioned BMDMs after treatment with PBS, MPs, Man-MPs, free Met, Met@MPs or Met@Man-MPs derived from BMDMs cells at the Met concentration of 20 μg mL^{-1}, or high concentration of Met at 200 μg mL^{-1} for 24 h by real time RT-PCR. Data are presented as means ± s.d. (n=4 biologically independent samples; one-way ANOVA followed by Tukey's HSD post-hoc test). Source data are provided as a Source Data file.

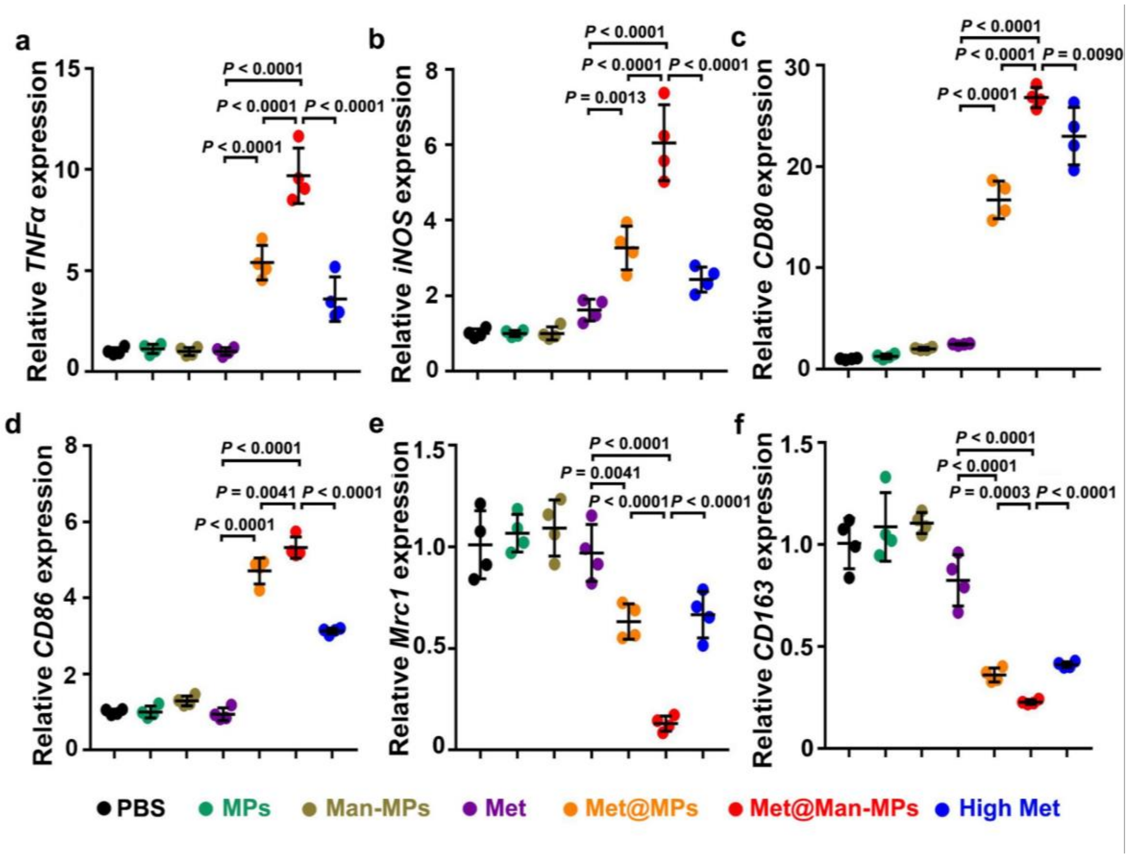

Supplementary Figure 14. Repolarization of M2-like macrophages to M1 phenotype by Met@Man-MPs derived from human THP-1-derived macrophages in IL-4-conditioned THP-1-derived macrophages.

(**a-f**) mRNA expression levels of *TNFα* (**a**), *iNOS* (**b**), *CD80* (**c**), *CD86* (**d**), *Mrc1* (**e**) and *CD163* (**f**) in IL-4-conditioned THP-1-derived macrophages after treatment with PBS, MPs, Man-MPs, free Met, Met@MPs or Met@Man-MPs derived from THP-1-derived macrophages at the Met concentration of 20 μg mL^{-1}, or high concentration of Met at 200 μg ml^{-1} for 24 h by real time RT-PCR. Data are presented as means ± s.d. (n=4 biologically independent samples; one-way ANOVA followed by Tukey's HSD post-hoc test). Source data are provided as a Source Data file.

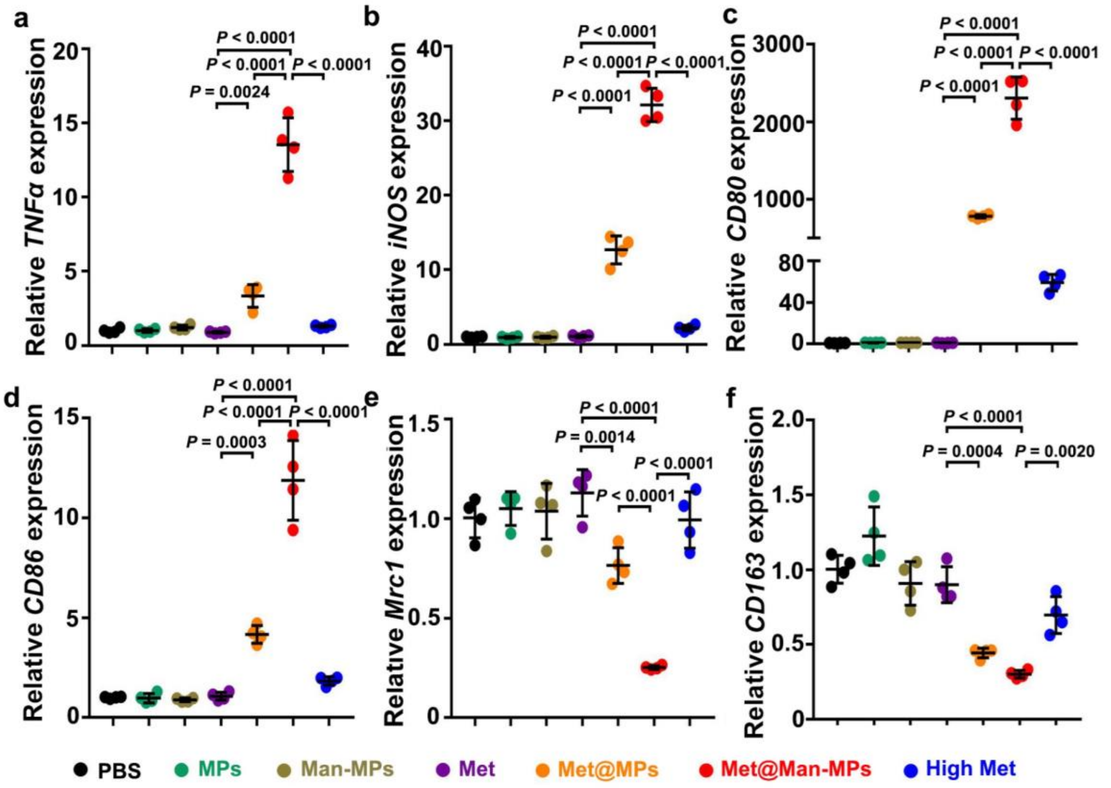

Supplementary Figure 15. Repolarization of M2-like macrophages to M1 phenotype by Met@Man-MPs derived from human MDMs in IL-4-conditioned MDMs.

(**a-f**) mRNA expression levels of *TNFα* (**a**), *iNOS* (**b**), *CD80* (**c**), *CD86* (**d**), *Mrc1* (**e**) and *CD163* (**f**) in IL-4-conditioned human MDMs after treatment with PBS, MPs, Man-MPs, free Met, Met@MPs or Met@Man-MPs derived from MDMs at the Met concentration of 20 μg mL^{-1}, or high concentration of Met at 200 μg ml^{-1} for 24 h by real time RT-PCR. Data are presented as means ± s.d. (n=4 biologically independent samples; one-way ANOVA followed by Tukey's HSD post-hoc test). Source data are provided as a Source Data file.

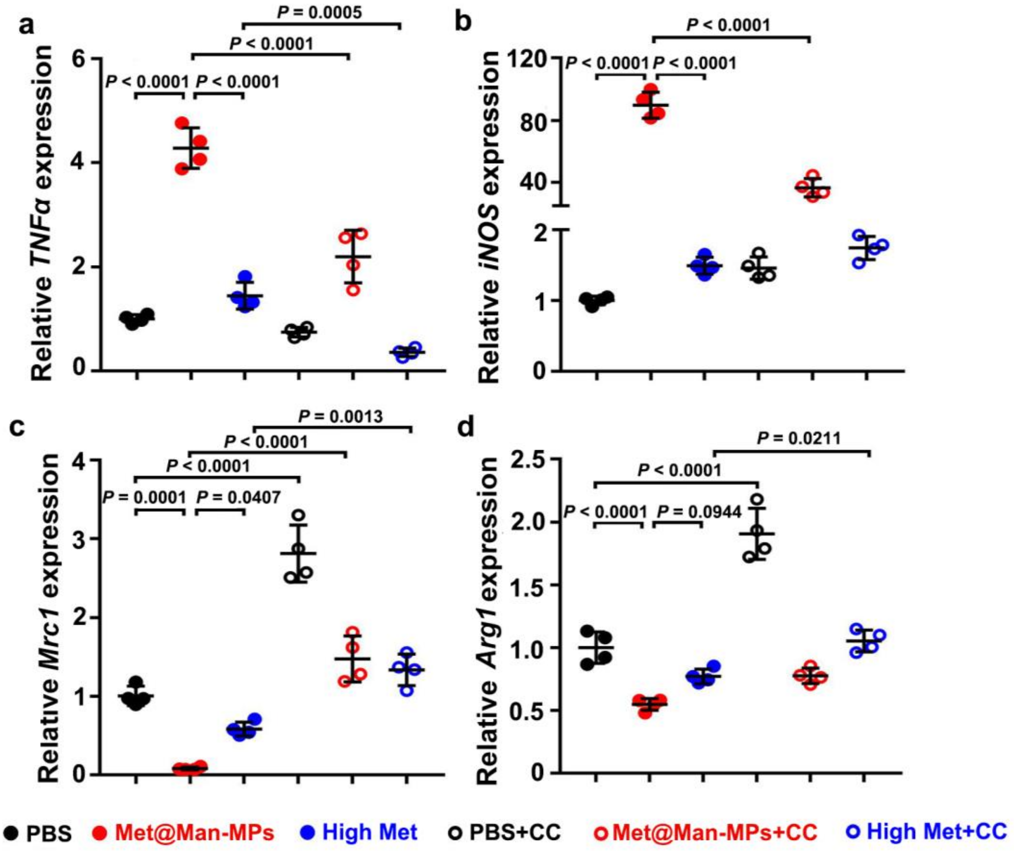

Supplementary Figure 16. Met@Man-MPs-induced repolarization of M2-like macrophages to M1 phenotype by AMPK signaling pathway.

(**a-d**) mRNA expression levels of *TNFα* (**a**), *iNOS* (**b**), *Mrc1* (**c**) and *Arg1* (**d**) in IL-4-conditioned RAW264.7 cells after treatment with PBS, Met@Man-MPs at the Met concentration of 20 μg mL^{-1}, or high concentration of Met at 200 μg ml^{-1} in the presence or absence 4 μM CC for 24 h by real time RT-PCR. Data are presented as means ± s.d. (n=4 biologically independent samples; one-way ANOVA followed by Tukey's HSD post-hoc test). Source data are provided as a Source Data file.

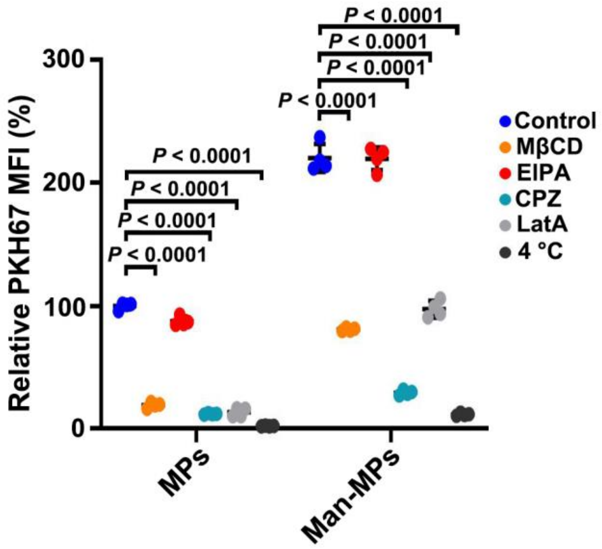

Supplementary Figure 17. Cellular uptake of MPs and Man-MPs in IL-4-conditioned RAW264.7 cells.

Intracellular PKH67 fluorescence intensity in IL-4-conditioned RAW264.7 cells after treatment with PKH67-labelled MPs or Man-MPs at the concentration of 10 μg protein mL^{-1} in the presence or absence of 5 mg/mL methyl-β-cyclodextrin (MβCD, an inhibitor of caveolin), 50 μg mL^{-1} 5-(*N*-ethyl-*N*-isopropyl) amiloride (EIPA, an inhibitor of macropinocytosis), 10 μg mL^{-1} chlorpromazine (CPZ, an inhibitor of clathrin) or 50 μM Lantraculin A (LatA, an inhibitor of phagocytosis) at 37 or 4 °C for 4 h by flow cytometry. Data are presented as means ± s.d. (n=4 biologically independent samples; one-way ANOVA followed by Tukey's HSD post-hoc test). Source data are provided as a Source Data file.

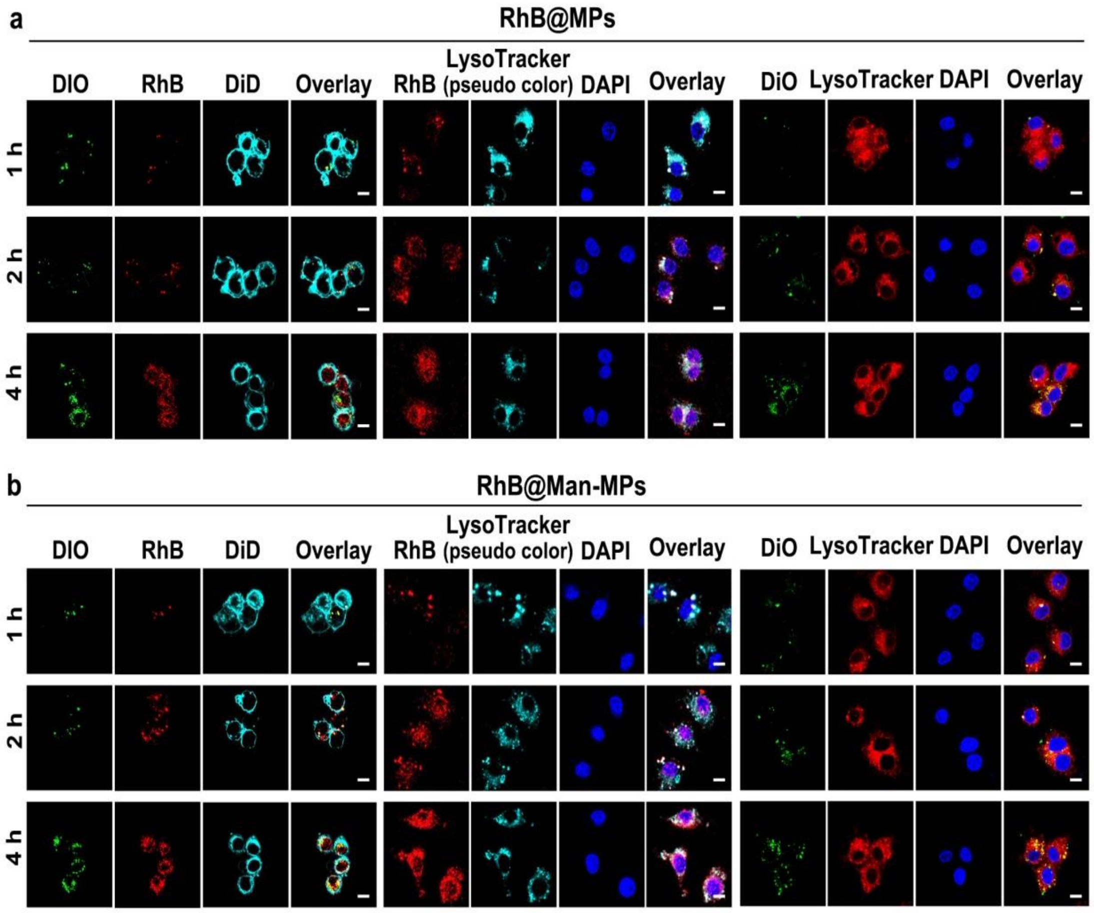

Supplementary Figure 18. Intracellular trafficking of RhB@MPs and RhB@Man-MPs in IL-4-conditioned RAW264.7 cells.

(**a,b**) Confocal microscopic images of IL-4-conditioned RAW264.7 cells after treatment with RhB-loaded DiO-labelled MPs (**a**) or Man-MPs (**b**) at the concentration of 10 μg protein mL^{-1} for different time intervals and then labelled with 1 μg mL^{-1} DiD (cell membrane labelling dye), 75 nM LysoTracker® Deep Red (lysosomes labelling dye) or 1 μg mL^{-1} DAPI (nucleus labelling dye), respectively. Images are representative of 3 independent experiments. Scale bars: 10 μm.

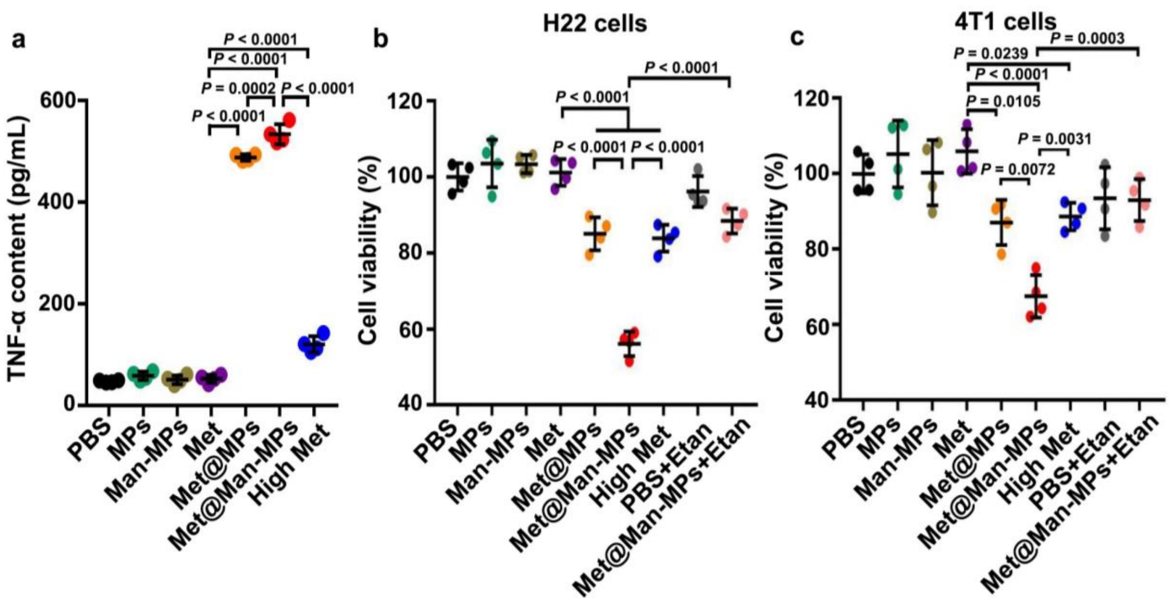

Supplementary Figure 19. Enhanced secretion of TNF-α involved in the cytotoxicity of the supernatants of Met@Man-MPs-educated IL-4-conditioned RAW264.7 cells against tumor cells.

(**a**) TNF-α content in the supernatants of IL-4-conditioned RAW264.7 cells at 24 h after treatment with PBS, MPs, Man-MPs, free Met, Met@MPs, Met@Man-MPs at the Met concentration of 20 μg mL^{-1}, or free Met at high concentration of 200 μg mL^{-1} by ELISA. Data are presented as means ± s.d. (n=4 biologically independent samples; one-way ANOVA followed by Tukey's HSD post-hoc test). (**b,c**) Cell cytotoxicity of the supernatants of IL-4-conditioned RAW264.7 cells treated with PBS, MPs, Man-MPs, free Met, Met@MPs, Met@Man-MPs at the Met concentration of 20 μg mL^{-1}, or free Met at high concentration of 200 μg mL^{-1} in the presence or absence of 0.5 μg mL^{-1} Etan against H22 (**b**) and 4T1 cells (**c**) for 24 h by CCK-8 assay. Data are presented as means ± s.d. (n=4 biologically independent samples; one-way ANOVA followed by Tukey's HSD post-hoc test). Source data are provided as a Source Data file.

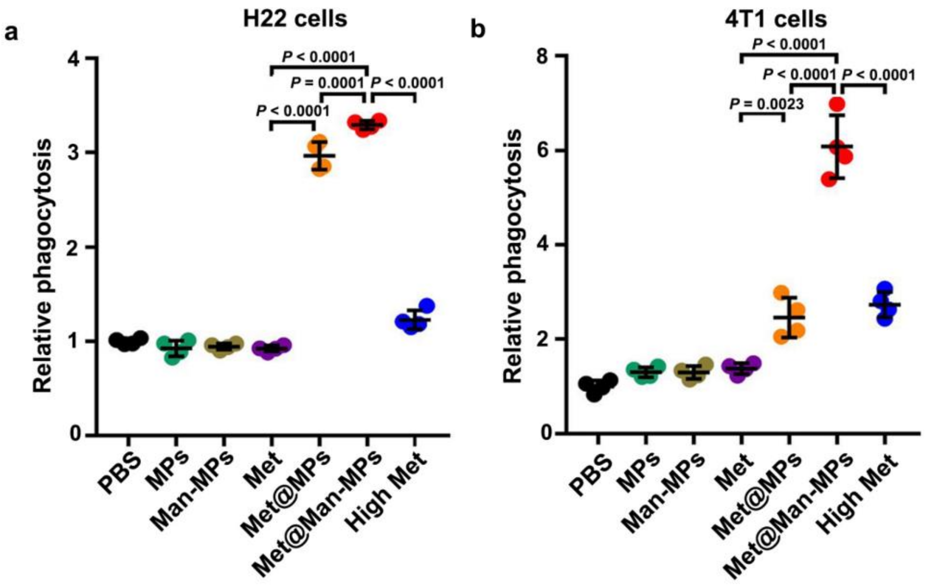

Supplementary Figure 20. Phagocytosis of tumor cells by Met@Man-MPs-treated IL-4 conditioned RAW264.7 cells.

(**a,b**) Relative phagocytosis of DiD-labelled H22 (**a**) and 4T1 cells (**b**) by IL-4-conditioned CFSE-labelled RAW264.7 cells treated with PBS, MPs, Man-MPs, free Met, Met@MPs, Met@Man-MPs at the Met concentration of 20 μg mL^{-1}, or free Met at high concentration of 200 μg mL^{-1} after co-culture at a ratio of 1:1 at 37 °C for 3 h by flow cytometry. Data are presented as means ± s.d. (n=4 biologically independent samples; one-way ANOVA followed by Tukey's HSD post-hoc test). Source data are provided as a Source Data file.

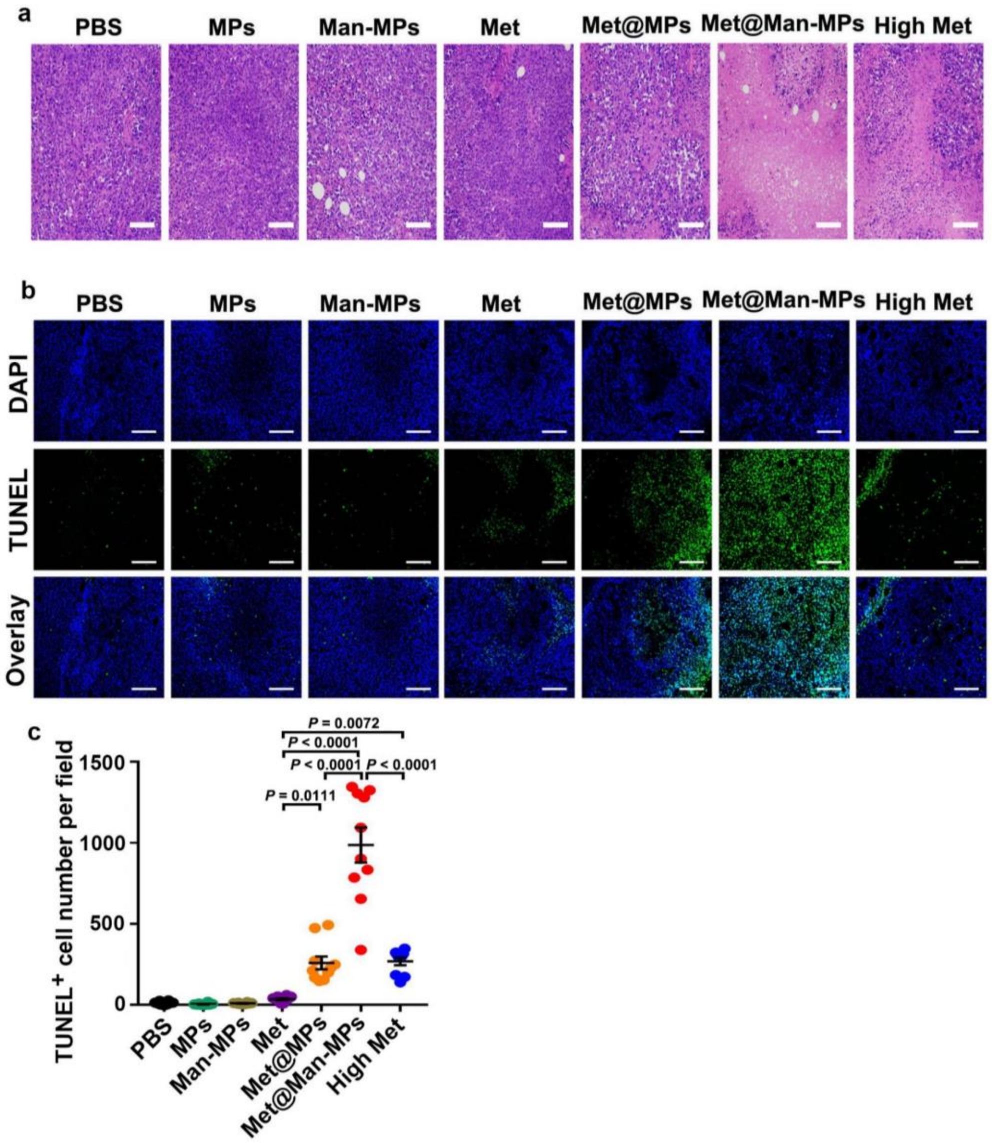

Supplementary Figure 21. H&E and TUNEL staining of tumor tissues in H22 tumor-bearing mice after treatment with Met@Man-MPs.

(a,b) Representative H&E (**a**) and TUNEL (**b**) staining images of tumor sections in H22 tumor-bearing mice after intravenous injection of PBS, MPs, Man-MPs, free Met, Met@MPs or Met@Man-MPs at the Met dosage of 10 mg kg^{-1}, or high dosage of Met at 100 mg kg^{-1} every two days for 6 times. Scale bars: 50 μm. Images are representative of 3 biologically independent experiments. (**c**) Quantification of TUNEL-positive cells in tumor tissues at the end of treatment. Data are presented as mean ± s.d. (n=10 fields for 3 mice; one-way ANOVA followed by Tukey's HSD post-hoc test). Source data are provided as a Source Data file.

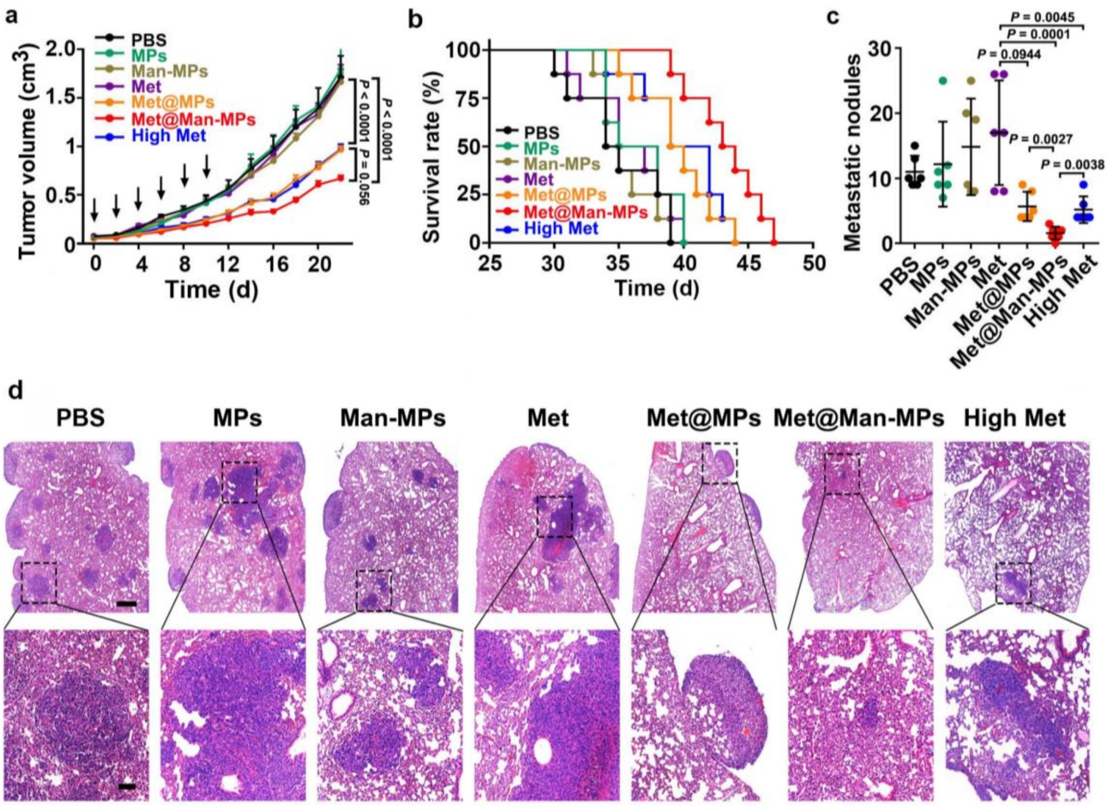

Supplementary Figure 22. Anticancer activity of Met@Man-MPs in 4T1 tumor-bearing mice.

(**a**) Tumor growth curves of 4T1 tumor-bearing mice after intravenous injection of PBS, MPs, Man-MPs, free Met, Met@MPs or Met@Man-MPs at the Met dosage of 10 mg kg^{-1}, or high dosage of Met at 100 mg kg^{-1} every two days for 6 times. The black arrows indicate the injection time. Data are presented as mean ± s.e.m. (n=6 mice per group; two-way ANOVA followed by Bonferroni's multiple comparisons post-test). (**b**) Kaplan-Meier survival plot of 4T1 tumor-bearing mice after treatment indicated in **a** (n=8 mice per group); (**c**) Metastatic nodule numbers in lungs of 4T1 tumor-bearing mice after treatment indicated in **a**. Data are presented as mean ± s.d. (n=6 mice per group; one-way ANOVA followed by Tukey's HSD post-hoc test). (**d**) H&E staining of lungs in 4T1 tumor-bearing mice after treatment indicated in **a.** The below is the amplification of the insets. Images are representative of 6 biologically independent mice. Scale bars: 500 μm (upper) and 100 μm (below). Source data are provided as a Source Data file.

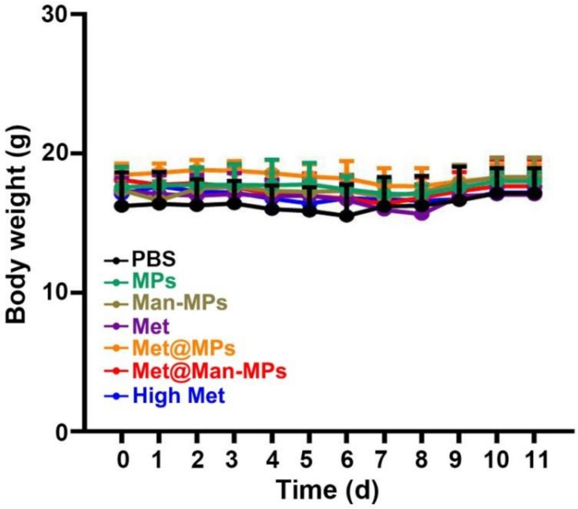

Supplementary Figure 23. Body weight of H22 tumor-bearing mice after treatment with Met@Man-MPs.

H22 tumor-bearing mice were intravenously injected with PBS, MPs, Man-MPs, free Met, Met@MPs or Met@Man-MPs at the Met dosage of 10 mg kg^{-1}, or high dosage of Met at 100 mg kg^{-1} every two days for 6 times, and the body weight was measured every day. Data are presented as mean ± s.d. (n=5 mice per group). Source data are provided as a Source Data file.

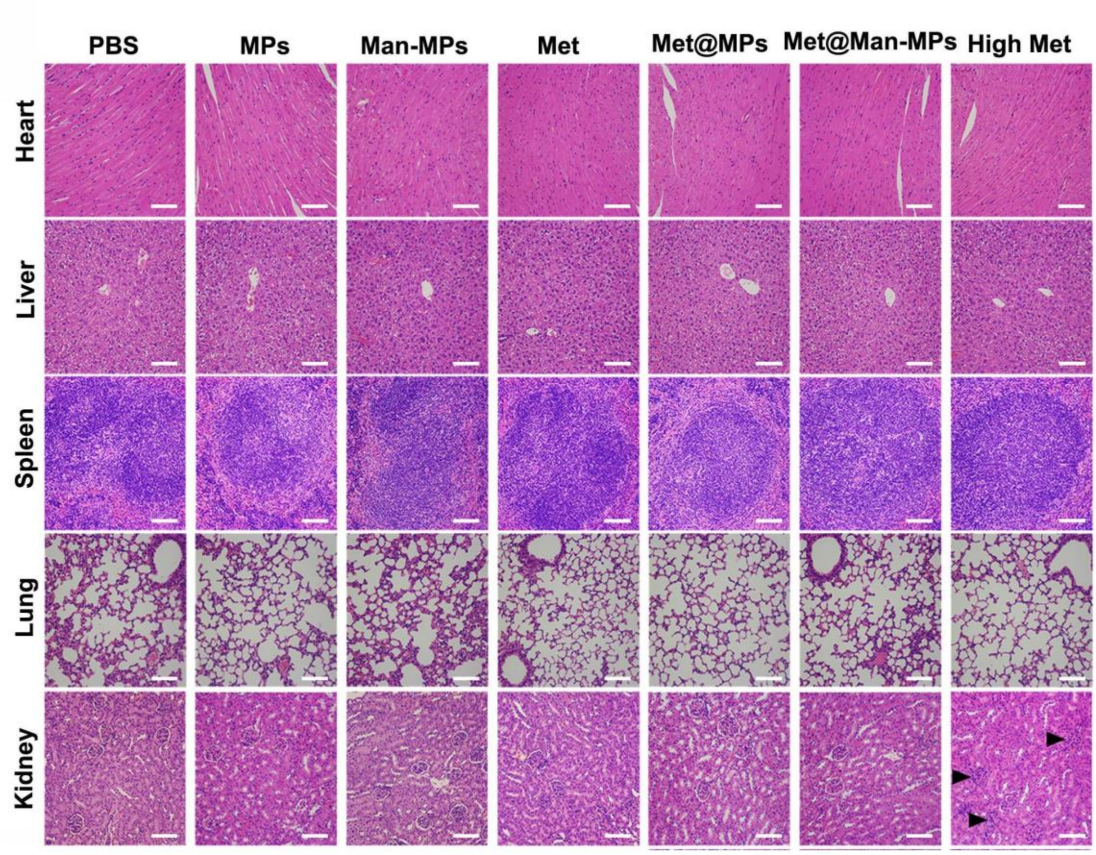

Supplementary Figure 24. Histological observation of tissues in H22 tumor-bearing mice after treatment with Met@Man-MPs.

H22 tumor-bearing mice were intravenously injected with PBS, MPs, Man-MPs, free Met, Met@MPs or Met@Man-MPs at the Met dosage of 10 mg kg^{-1}, or high dosage of Met at 100 mg kg^{-1} every two days for 6 times. The histological sections of tissues were stained by H&E. The black arrows show the injured glomerular. Images are representative of 3 biologically independent mice. Scale bars: 50 μm.

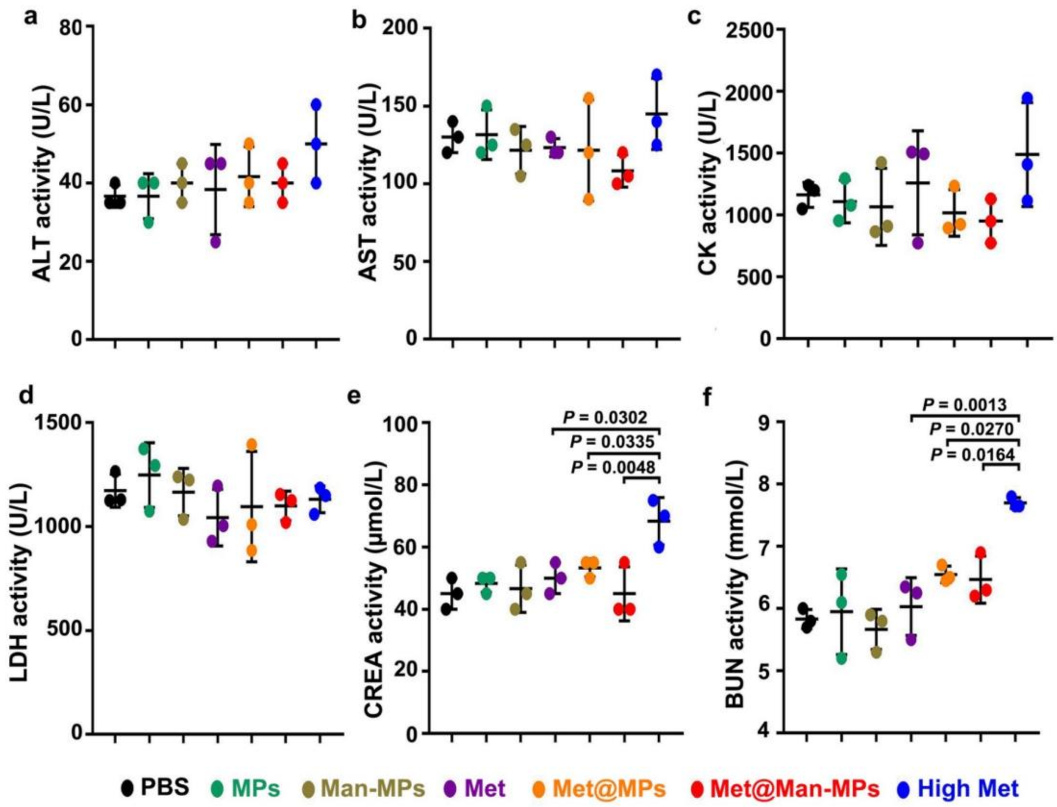

Supplementary Figure 25. Serological analysis of H22 tumor-bearing mice after treatment with Met@Man-MPs.

(**a-e**) The serological analysis of alanine aminotransferase (ALT, **a**), aspartate aminotransferase (AST, **b**), creatine kinase (CK, **c**), lactate dehydrogenase (LDH, **d**), creatinine (CREA, **e**) and blood urea nitrogen (BUN, **f**) in H22 tumor-bearing mice intravenously injected with PBS, MPs, Man-MPs, free Met, Met@MPs or Met@Man-MPs at the Met dosage of 10 mg kg^{-1}, or high dosage of Met at 100 mg kg^{-1} every two days for 6 times. Data are presented as mean ± s.d. (n=3 mice per group; one-way ANOVA followed by Tukey's HSD post-hoc test). Source data are provided as a Source Data file.

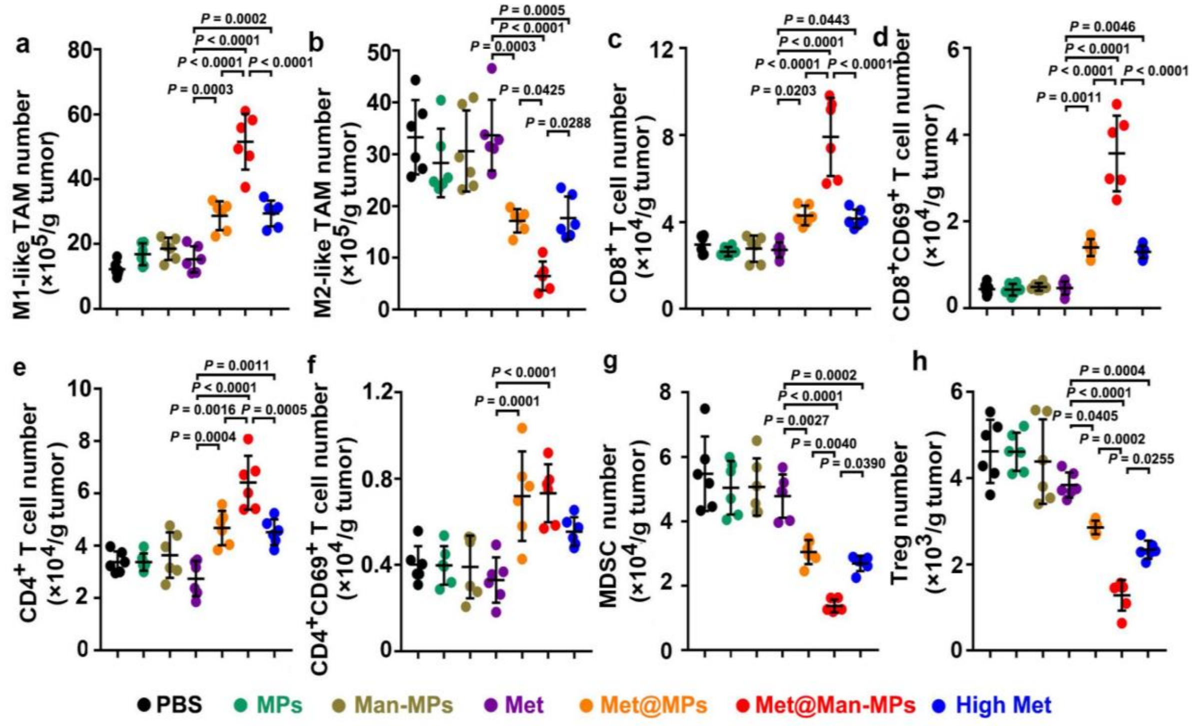

Supplementary Figure 26. Improved immune microenvironment of Met@Man-MPs in 4T1 tumor-bearing mice.

(**a**-**h**) The numbers of M1-like TAMs (**a**), M2-like TAMs (**b**), $CD8^+$ T cells (**c**), $CD8^+CD69^+$ T cells (**d**), $CD4^+$ T cells (**e**), $CD4^+CD69^+$ T cells (**f**), MDSCs (**g**) and Tregs (**h**) in tumor tissues of 4T1 tumor-bearing mice after intravenous injection of free Met, Met@MPs or Met@Man-MPs at Met dosage of 10 mg kg^{-1}, or high dosage of Met at 100 mg kg^{-1} every two days for 6 times. Data are presented as mean ± s.d. (n=6 mice per group; one-way ANOVA followed by Tukey's HSD post-hoc test). Source data are provided as a Source Data file.

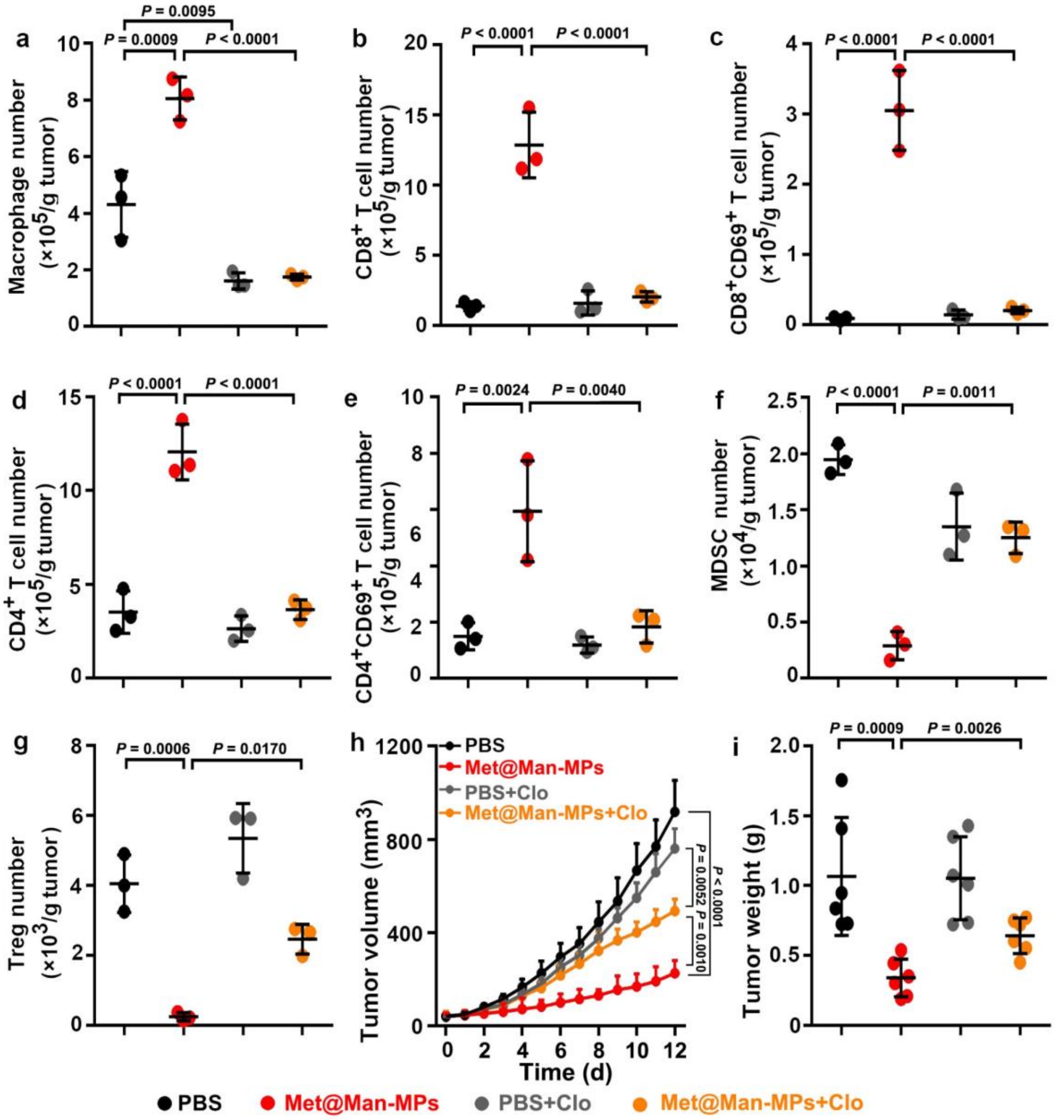

Supplementary Figure 27. Effects of macrophage depletion on the Met@Man-MPs-induced immune cell numbers in tumor tissues and anticancer activity in H22 tumor-bearing mice.

(**a-g**) The numbers of macrophages (**a**), $CD8^+$ T cells (**b**), $CD8^+CD69^+$ T cells (**c**), $CD4^+$ T cells (**d**), $CD4^+CD69^+$ T cells (**e**), MDSCs (**f**) and Tregs (**g**) in tumor tissues of H22 tumor-bearing mice after intraperitoneal injection of clodronate disodium liposomes at the dosage of 3.75 mg kg^{-1} every four days for 4 times, or/and intravenous injection of Met@Man-MPs at the Met dosage of 10 mg kg^{-1} every two days for 6 times. Data are presented as mean ± s.d. (n=3 mice per group; one-way

ANOVA followed by Tukey's HSD post-hoc test). (**h**) Tumor volume of H22 tumor-bearing mice after treatment as above. Data are presented as mean ± s.e.m. (n=6 mice per group; two-way ANOVA followed by Bonferroni's multiple comparisons post-test). (**i**) Tumor weight of H22 tumor-bearing mice after treatment as above. Data are presented as mean ± s.d. (n=6 mice per group; one-way ANOVA followed by Tukey's HSD post-hoc test). Source data are provided as a Source Data file.

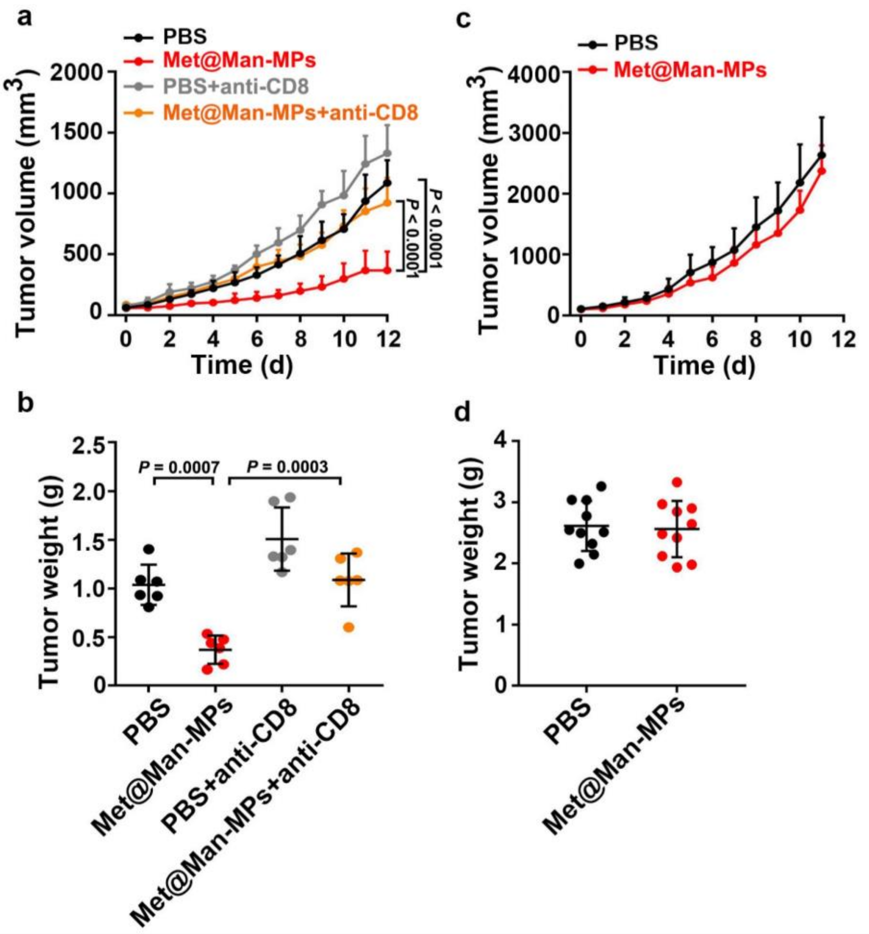

Supplementary Figure 28. CD8^{+} T cell-involved in the anticancer activity of Met@Man-MPs.

(**a,b**) Tumor volume (**a**) and weight (**b**) of H22 tumor-bearing mice after intraperitoneal injection of anti-CD8 antibody at the dosage of 100 μg per mice every three days for 5 times and intravenous injection of Met@Man-MPs at the Met dosage of 10 mg kg^{-1} every two days for 6 times. Data are presented as mean ± s.e.m for **a** and mean ± s.d. for **b**. (n=6 mice per group; two-way ANOVA followed by Bonferroni's multiple comparisons post-test for **a**; one-way ANOVA followed by Tukey's HSD post-hoc test for **b**). (**c,d**) Tumor volume (**c**) and weight (**d**) of nude mice bearing H22 tumors after intravenous injection of Met@Man-MPs at the Met dosage of 10 mg kg^{-1} every two days for 6 times. Data are presented as mean ± s.e.m for **c** and mean ± s.d. for **d** (n=10 mice per group). Source data are provided as a Source Data file.

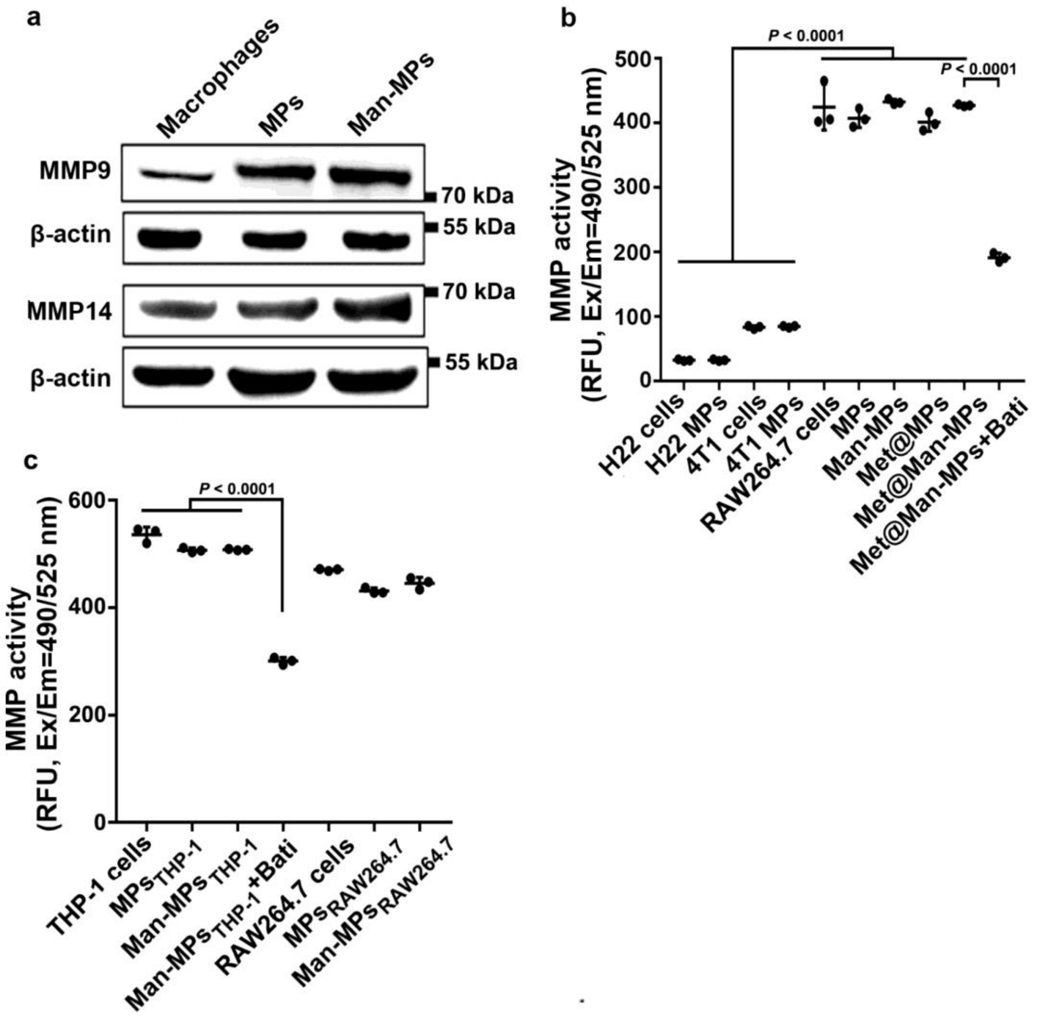

Supplementary Figure 29. MMP activity of Man-MPs.

(a) MMP9 and MMP14 expression in RAW264.7 cells, MPs and Man-MPs originated from RAW264.7 cells by western blot. Images are representative of 3 independent experiments. (**b**) MMP activity in H22 cells, H22 cell-originated MPs, 4T1 cells, 4T1 cell-originated MPs, RAW264.7 macrophages, RAW264.7 macrophage-originated MPs, Man-MPs, Met@MPs and Met@Man-MPs pretreated with or without 0.6 mg mL^{-1} Bati measured by MMP activity assay kit. Data are presented as mean ± s.d. (n=3 biologically independent samples; one-way ANOVA followed by Tukey's HSD post-hoc test). (**c**) MMP activity in RAW264.7 macrophages, THP-1-derived macrophages, RAW264.7 macrophage-originated MPs and Man-MPs ($MPs_{RAW164.7}$

and Man-$MPs_{RAW264.7}$, respectively), THP-1-derived macrophage-originated MPs and Man-MPs (MPs_{THP-1} and Man-MPs_{THP-1}, respectively) pretreated with or without 0.6 mg mL^{-1} Bati measured by MMP activity assay kit. Data are presented as mean ± s.d. (n=3 biologically independent samples; one-way ANOVA followed by Tukey's HSD post-hoc test). Source data are provided as a Source Data file.

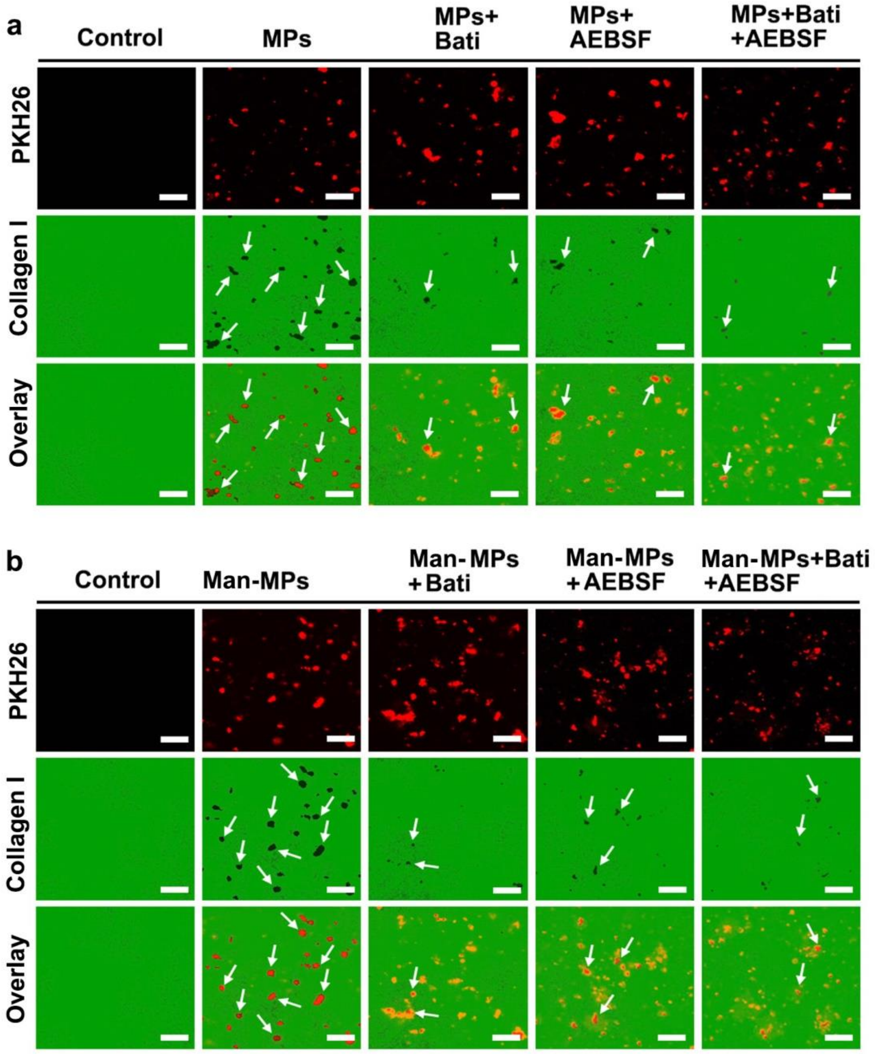

Supplementary Figure 30. Collagen I degradation by Man-MPs in MMP-dependent manner.

(**a,b**) Colocalization of MPs (**a**) or Man-MPs (**b**) and collagen I films after biotinylated collagen I films were incubated with PKH26-labelled MPs or Man-MPs at the concentrations of 50 μg protein mL^{-1} pretreated with or without 0.6 mg mL^{-1} Bati or 100 μg mL^{-1} AEBSF for 72 h and then stained with streptavidin-FITC by confocal microscopy. Images are representative of 3 independent experiments. Scale bars: 2 μm.

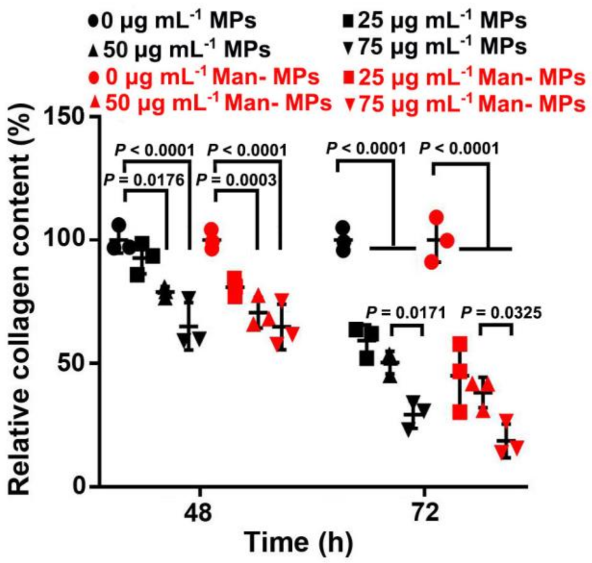

Supplementary Figure 31. Collagen I degradation by Man-MPs in a concentration-dependent manner.

Relative collagen content at different time intervals after treatment with different concentrations of MPs or Man-MPs measured by Sirius red total collagen detection kit. Data are presented as mean ± s.d. (n=3 biologically independent samples; one-way ANOVA followed by Tukey's HSD post-hoc test). Source data are provided as a Source Data file.

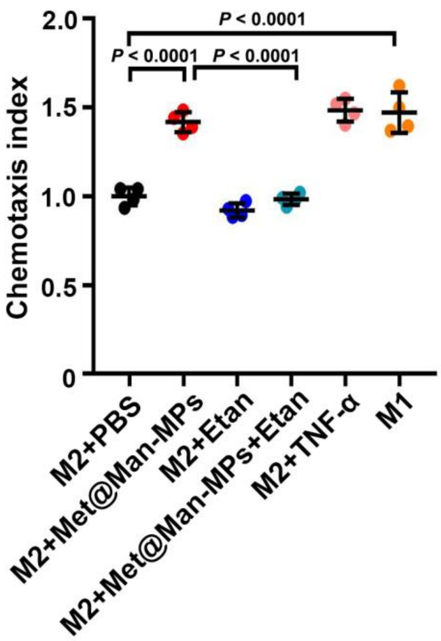

Supplementary Figure 32. Chemotaxis activity of $CD8^+$ T cells by Met@Man-MPs-reset macrophages.

Lymphocytes isolated from BALB/c mice were seeded in the top chambers and IL-4-conditioned RAW264.7 cells pretreated with PBS, Met@Man-MPs, Etan, or combination of Met@Man-MPs and Etan at the Met concentration of 20 μg mL^{-1} and Etan concentration of 0.5 μg mL^{-1} for 24 h were added in the bottom chambers. After 8 h, the numbers of $CD45^+CD3^+CD8^+$ T cells in the bottom chambers were recorded by flow cytometry. IL-4-conditioned RAW264.7 cells supplemented with TNF-α (0.25 ng mL^{-1}), and LPS- and IFN-γ-conditioned RAW264.7 cells (M1-like macrophages) were used as positive controls. Data are presented as mean ± s.d. (n=4 biologically independent samples; one-way ANOVA followed by Tukey's HSD post-hoc test). Source data are provided as a Source Data file.

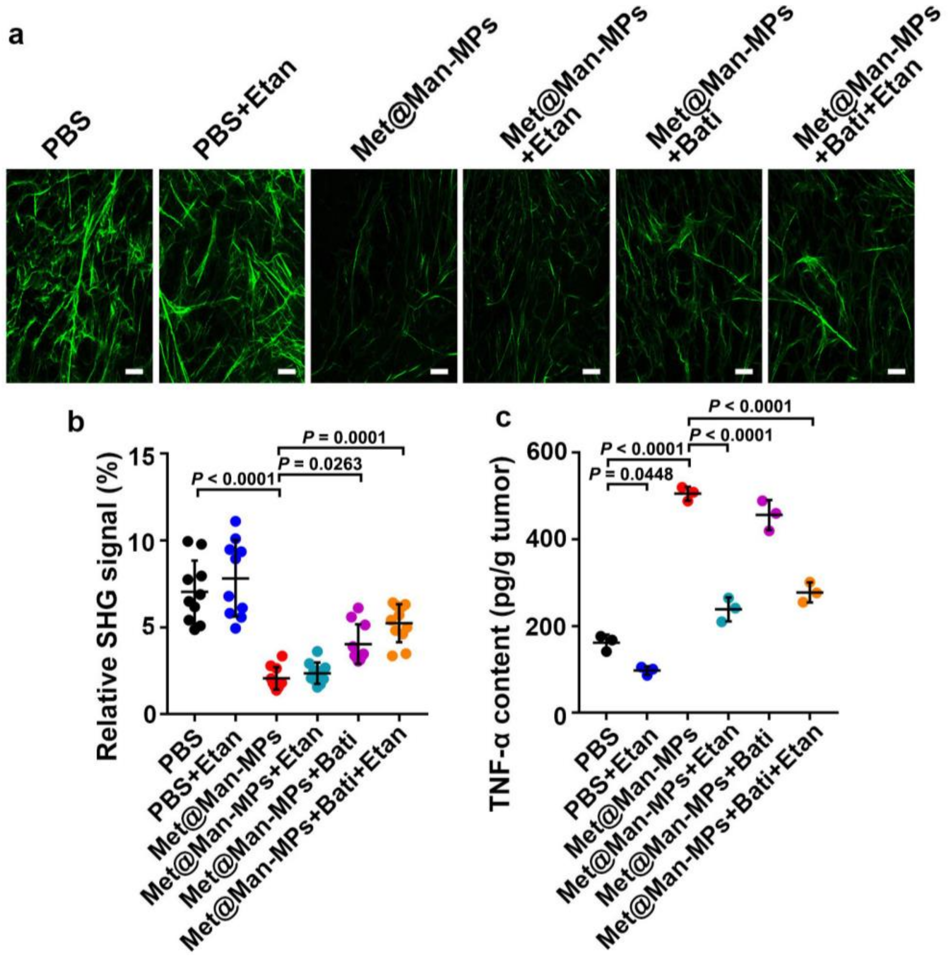

Supplementary Figure 33. Collagen degradation and TNF-α content after treatment with Bati-pretreated Met@Man-MPs or Etan.

(**a**) SHG imaging of tumor tissues of H22 tumor-bearing mice after intravenous injection of Met@Man-MPs pretreated with or without Bati (0.6 mg mL^{-1}) at the Met dosage of 10 mg kg^{-1} every two days for 6 times, or/and intraperitoneal injection of Etan at dosage of 5 mg kg^{-1} every four days for 4 times. Images are representative of 3 biologically independent mice. Scale bars: 50 μm. (**b)** Relative SHG signal intensity in tumor tissues of H22 tumor-bearing mice after treatment indicated in **a**. Data are presented as mean ± s.d. (n=10 fields for 3 mice; one-way ANOVA followed by Tukey's HSD post-hoc test). (**c**) TNF-α contents in tumor tissues of H22 tumor-bearing mice after treatment indicated in **a**. Data are presented as mean ± s.d. (n=3 mice per group; one-way ANOVA followed by Tukey's HSD post-hoc test). Source data are provided as a Source Data file.

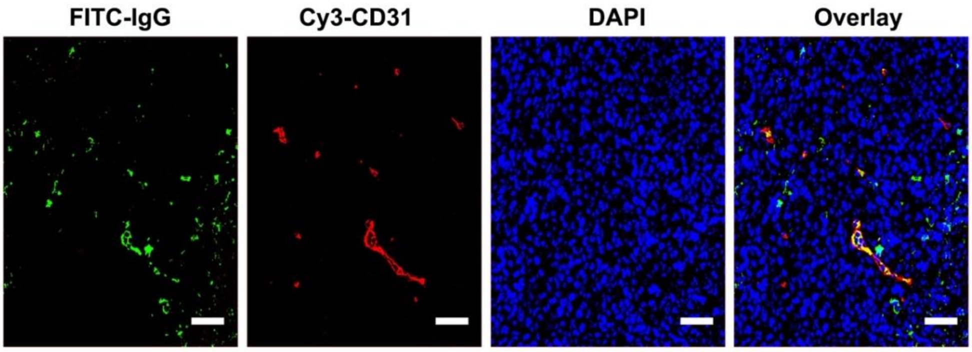

Supplementary Figure 34. Distribution of endogenous IgG in tumor tissues.

Colocalization of endogenous IgG labelled with FITC-conjugated IgG antibody (green) with endothelial cells labelled with Cy3-conjugated CD31 antibody (red) in tumor sections of H22 tumor-bearing mice after treatment with Cy5-labelled anti-PD-1 antibody (100 μg per mice). The nuclei were stained with DAPI (blue). Images are representative of 3 independent experiments. Scale bars: 50 μm.

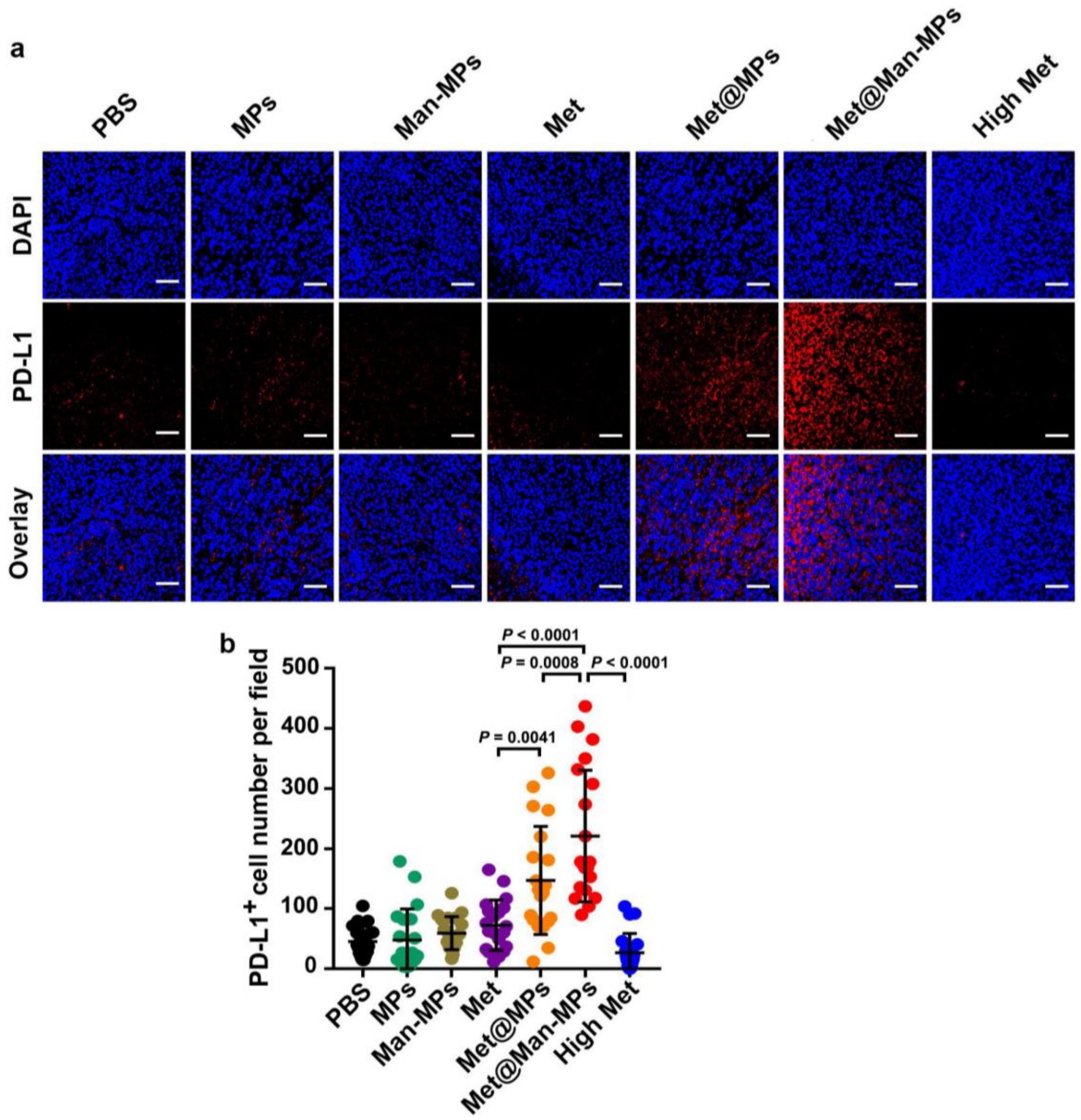

Supplementary Figure 35. PD-L1 expression after treatment with Met@Man-MPs.

(**a**) Immunofluorescence staining of PD-L1 expression in tumors of H22 tumor-bearing mice after intravenous injection of PBS, MPs, Man-MPs, free Met, Met@MPs or Met@Man-MPs at the Met dosage of 10 mg kg^{-1}, or high dosage of Met at 100 mg kg^{-1} every two days for 6 times. Images are representative of 3 biologically independent mice. Scale bars: 50 µm. (**b**) PD-L1^{+} cell number in tumors of H22 tumor-bearing mice after treatment indicated in **a**. Data are presented as mean ± s.d. (n=20 fields for 3 mice; one-way ANOVA followed by Tukey's HSD post-hoc test). Source data are provided as a Source Data file.

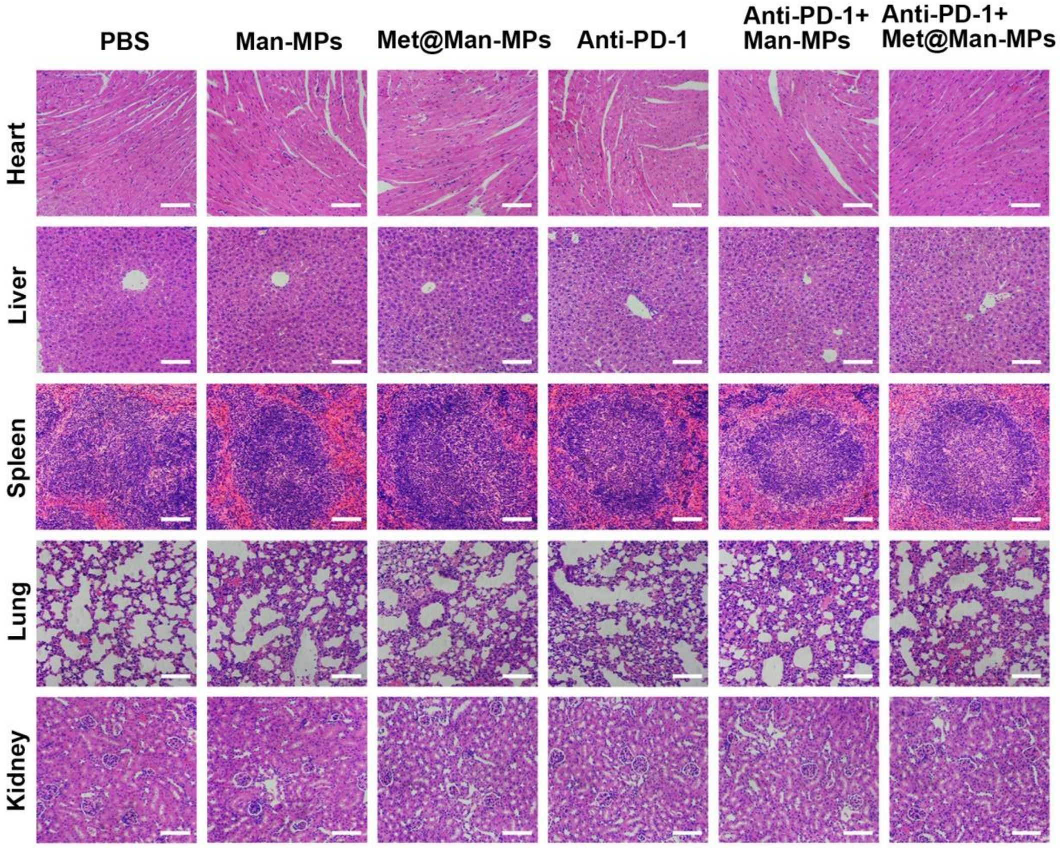

Supplementary Figure 36. Histological observation of tissues in H22 tumor-bearing mice after treatment with anti-PD-1 antibody and Met@Man-MPs.

H22 tumor-bearing mice were intravenously injected with PBS, Man-MPs, Met@Man-MPs, anti-PD-1 antibody, combination of Man-MPs and anti-PD-1 antibody, or combination of Met@Man-MPs and anti-PD-1 antibody at the anti-PD-1 antibody dosage of 100 μg per mouse and Met dosage of 10 mg kg^{-1}. Man-MPs or Met@Man-MPs were intravenously injected at day 0, 2, 4, 6, 8 and 10, and anti-PD-1 antibody was intraperitoneally injected at day 1, 5 and 9. Images are representative of 6 biologically independent mice. Scale bars: 50 μm.

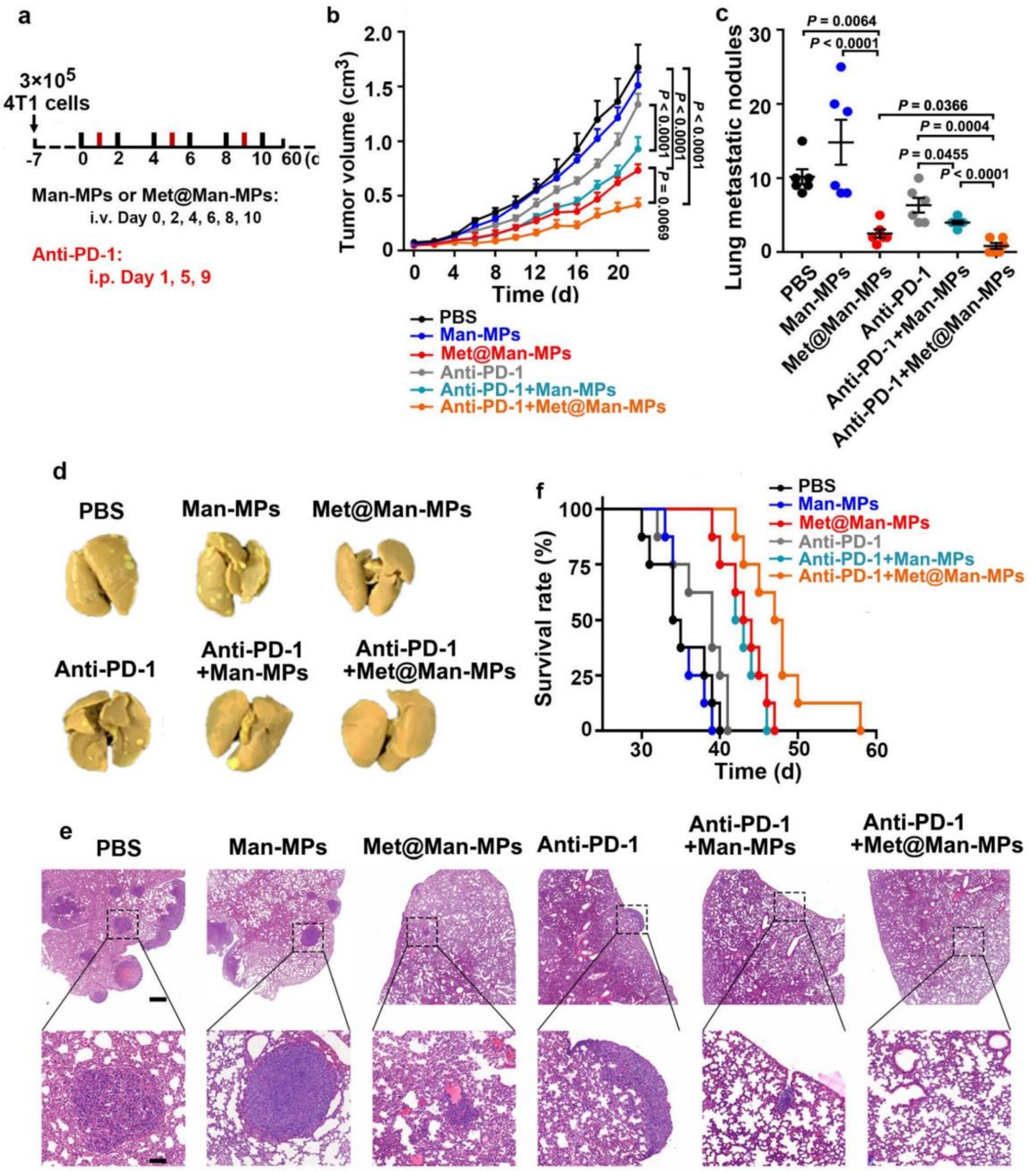

Supplementary Figure 37. Anticancer activity of combination of anti-PD-1 antibody and Met@Man-MPs in 4T1 tumor-bearing mice.

(a) Schematic schedule for anticancer experiment in 4T1 tumor-bearing mice. **(b)** Tumor growth curves of 4T1 tumor-bearing mice after treatment with PBS, Man-MPs, Met@Man-MPs, anti-PD-1 antibody, combination of Man-MPs and anti-PD-1 antibody, or combination of Met@Man-MPs and anti-PD-1 antibody at the anti-PD-1

antibody dosage of 100 μg per mouse and Met dosage of 10 mg kg^{-1} indicated in **a**. Data are presented as mean ± s.e.m. (n=6 mice per group; two-way ANOVA followed by Bonferroni's multiple comparisons post-test). (**c**) Metastatic nodule numbers in lungs of 4T1 tumor-bearing mice after treatment indicated in **a**. Data are presented as mean ± s.d. (n=6 mice per group; one-way ANOVA followed by Tukey's HSD post-hoc test). (**d)** Lung images of 4T1 tumor-bearing mice after treatment indicated in **a**. Images are representative of 6 biologically independent mice. (**e**) H&E staining of lungs in 4T1 tumor-bearing mice after treatment indicated in **a**. The below is the amplification of the insets. Images are representative of 6 biologically independent mice. Scale bars: 500 μm (upper) and 100 μm (below). (**f**) Kaplan-Meier survival plot of 4T1 tumor-bearing mice after treatment indicated in **a** (n=8 mice per group). Source data are provided as a Source Data file.

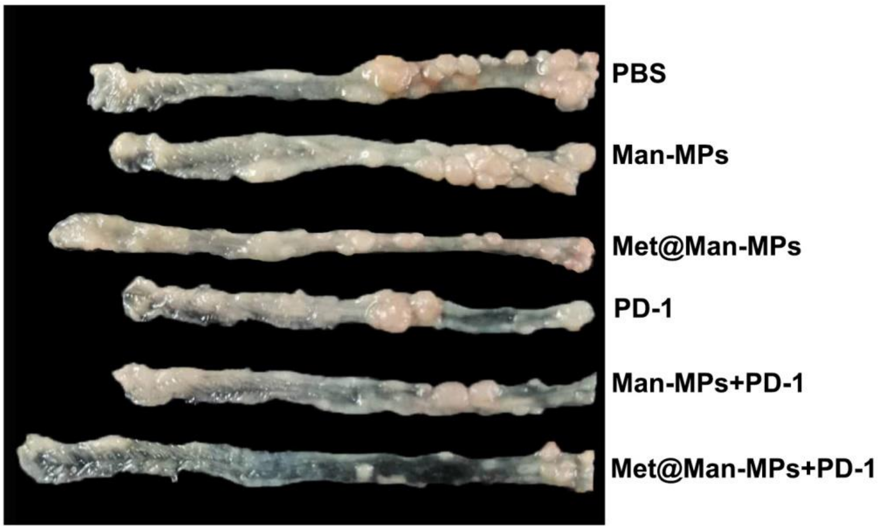

Supplementary Figure 38. Anticancer activity of combination of anti-PD-1 antibody and Met@Man-MPs in AOM/DSS-induced CAC mice.

Colon tumors from CAC mice after intravenous injection of PBS, Man-MPs, Met@Man-MPs at the Met dosage of 10 mg kg^{-1} every 3 days for 8 times in the presence or absence of intraperitoneal injection of anti-PD-1 antibody at the dosage of 100 μg per mouse every 4 days for 6 times. Images are representative of 5 biologically independent mice.

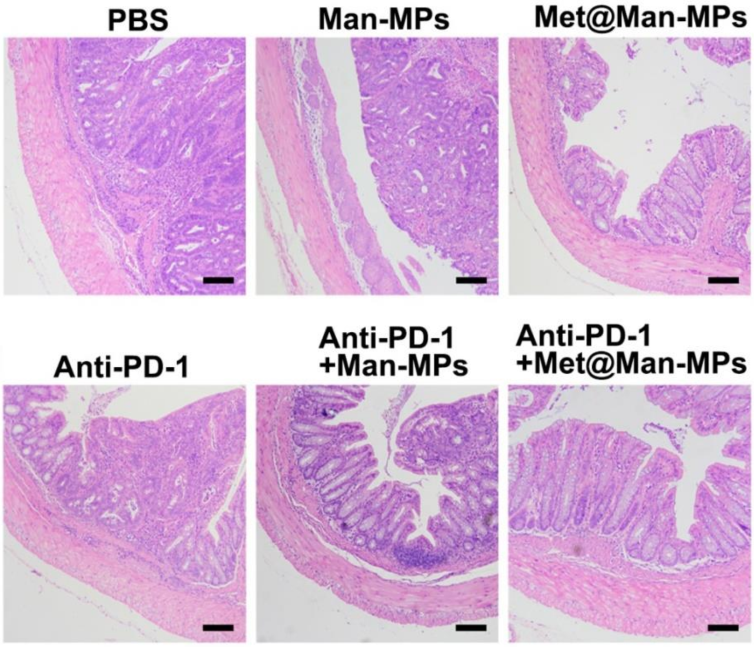

Supplementary Figure 39. Histological observation of colon tissues in AOM/DSS-induced CAC mice after treatment with combination of anti-PD-1 antibody and Met@Man-MPs.

H&E-stained sections of colon tumors from CAC mice after intravenous injection of PBS, Man-MPs, Met@Man-MPs at the Met dosage of 10 mg kg^{-1} every 3 days for 8 times in the presence or absence of intraperitoneal injection of anti-PD-1 antibody at the dosage of 100 μg per mouse every 4 days for 6 times. Images are representative of 5 biologically independent mice. Scale bars: 50 μm.

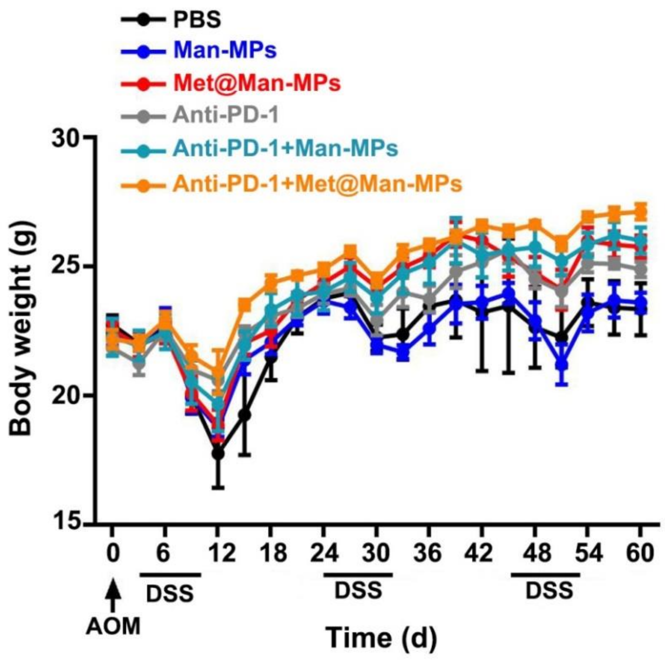

Supplementary Figure 40. Body weight changes of AOM/DSS-induced CAC mice throughout the entire anticancer study. Data are presented as mean ± s.d. (n=5 mice per group). Source data are provided as a Source Data file.

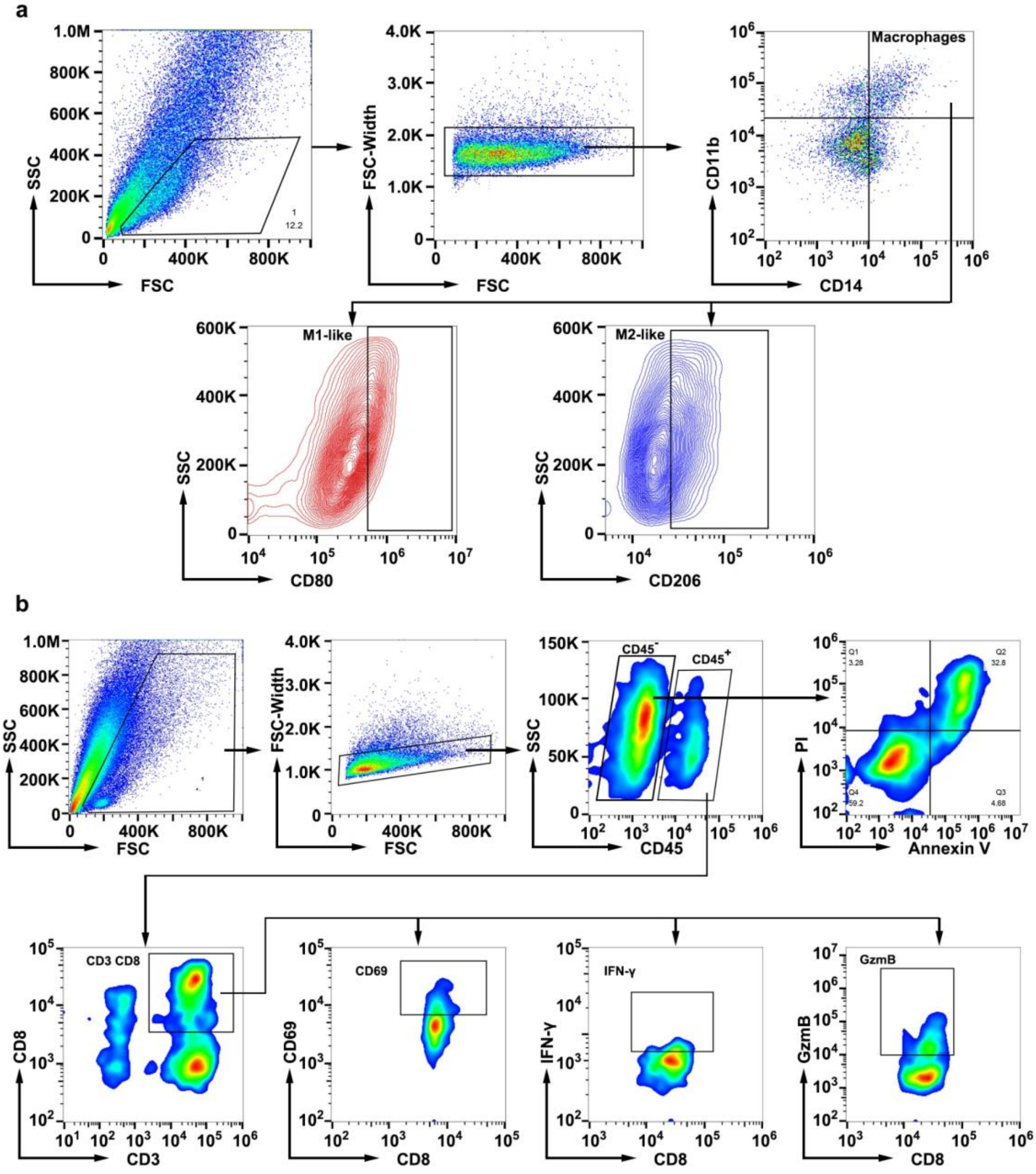

Supplementary Figure 41. Gating strategy for identifying M1- and M2-like TAMs in organotypic slices from the liver cancer patient-derived tumors.

(**a**) Gating strategy for identifying M1- and M2-like TAMs in organotypic slices from the liver cancer patient-derived tumors presented on Fig. 10b,c and Supplementary Fig. 42b,c. (**b**) Gating strategy for identifying apoptosis tumor cells, $CD8^+$ T cells, $CD8^+CD69^+$ T cells, $CD8^+$IFN-γ^+ T cells, $CD8^+GzmB^+$ T cells in organotypic slices from the liver cancer patient-derived tumors presented on Fig. 10a, 10d-g and Supplementary Fig. 42a, 42d-g.

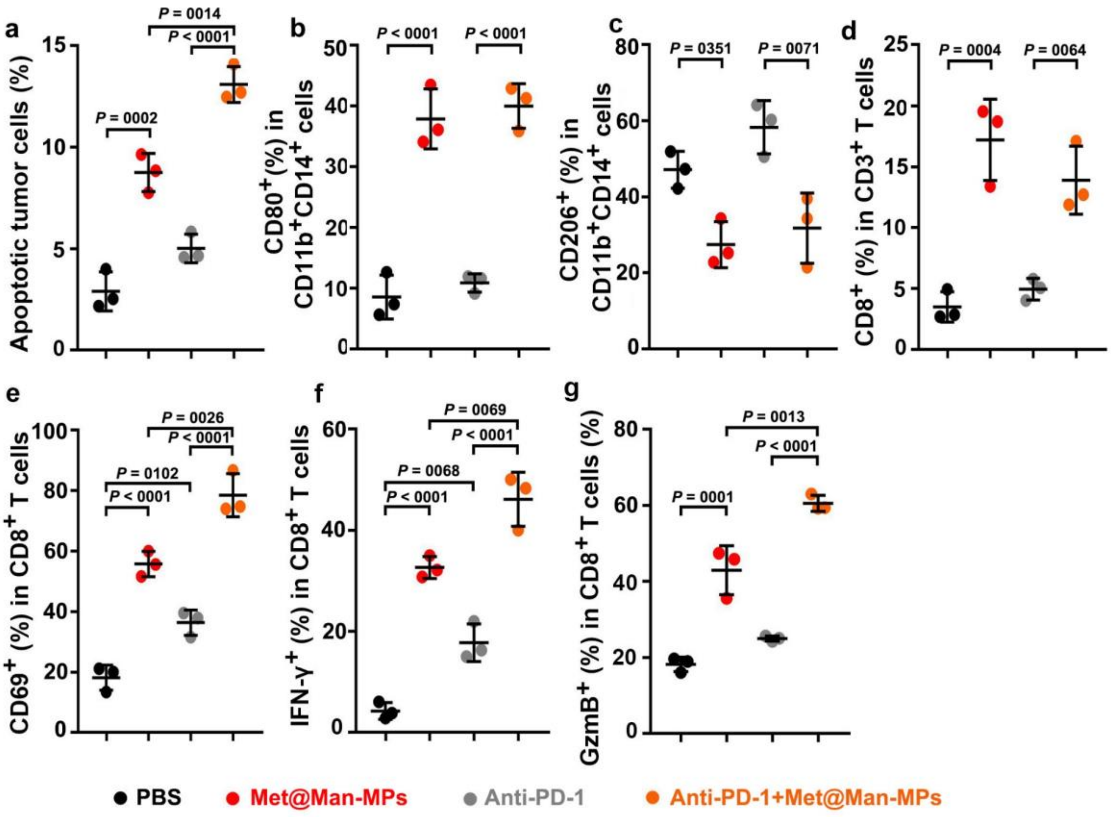

Supplementary Figure 42. Apoptosis and improved tumor immune microenvironment induced by combination of anti-PD-1 antibody and Met@Man-MPs in organotypic slices from another liver cancer patient-derived tumor.

(**a**) The ratio of apoptotic tumor cells after the tumor slices from liver cancer patient-derived tumor were treated with anti-human PD-1 antibody, Met@Man-MPs or Met@Man-MPs plus anti-human PD-1 antibody at the concentration of anti-PD-1 antibody and Met of 20 and 40 μg mL^{-1} in the presence of PBMCs, respectively for 36 h. (**b-g**) Percentages of M1-like TAMs (**b**), M2-like TAMs (**c**), $CD8^+$ T (**d**), $CD8^+CD69^+$ T (**e**), $CD8^+IFN\text{-}\gamma^+$ T (**f**) and $CD8^+GzmB^+$ T cells (**g**) in the tumor slices from patient-derived tumor after treatment with anti-human PD-1 antibody, Met@Man-MPs or Met@Man-MPs plus anti-human PD-1 antibody at the concentration of anti-PD-1 antibody and Met of 20 and 40 μg mL^{-1} in the presence of PBMCs, respectively for 36 h. Data are presented as mean ± s.d. (n=3 biologically independent samples; one-way ANOVA followed by Tukey's HSD post-hoc test). Source data are provided as a Source Data file.
