# Supplementary material for: Boosting anti-PD-1 therapy with metformin-loaded macrophage-derived microparticles
Source: Nat Commun. 2021 Jan 19;12:440. doi: 10.1038/s41467-020-20723-x (PMC7815730; doi:10.1038/s41467-020-20723-x)
Supplement: Supplementary file 3 — Reporting Summary [file 41467_2020_20723_MOESM3_ESM.pdf]

nature research

Corresponding author(s): Lu Gan, Bo Huang, Xiangliang Yang

Last updated by author(s): Nov 28, 2020

Reporting Summary

Nature Research wishes to improve the reproducibility of the work that we publish. This form provides structure for consistency and transparency in reporting. For further information on Nature Research policies, see our Editorial Policies and the Editorial Policy Checklist.

Statistics

For all statistical analyses, confirm that the following items are present in the figure legend, table legend, main text, or Methods section.

n/a	Confirmed	
☐	☒	The exact sample size (*n*) for each experimental group/condition, given as a discrete number and unit of measurement
☐	☒	A statement on whether measurements were taken from distinct samples or whether the same sample was measured repeatedly
☐	☒	The statistical test(s) used AND whether they are one- or two-sided<br>*Only common tests should be described solely by name; describe more complex techniques in the Methods section.*
☒	☐	A description of all covariates tested
☒	☐	A description of any assumptions or corrections, such as tests of normality and adjustment for multiple comparisons
☐	☒	A full description of the statistical parameters including central tendency (e.g. means) or other basic estimates (e.g. regression coefficient) AND variation (e.g. standard deviation) or associated estimates of uncertainty (e.g. confidence intervals)
☐	☒	For null hypothesis testing, the test statistic (e.g. *F*, *t*, *r*) with confidence intervals, effect sizes, degrees of freedom and *P* value noted<br>*Give P values as exact values whenever suitable.*
☒	☐	For Bayesian analysis, information on the choice of priors and Markov chain Monte Carlo settings
☒	☐	For hierarchical and complex designs, identification of the appropriate level for tests and full reporting of outcomes
☒	☐	Estimates of effect sizes (e.g. Cohen's *d*, Pearson's *r*), indicating how they were calculated

Our web collection on statistics for biologists contains articles on many of the points above.

Software and code

Policy information about availability of computer code

Data collection	Flow-cytometry data were collected with CytExpert and CXP Cytometer. Images were collected with imaging softwares, such as Olympus FluoView31S (Ver.2.3.1.163) for confocal, Zen lite 2012 software (Ver.1.1.2.0) for tumor of dorsal window chamber and SHG, and Leica Application suite V3 for optical images.
Data analysis	CytExpert (Ver.2.3.0.84), CXP Cytometer 2.3 and Flowjo (Ver.10.5.3) were used to analyse flow-cytometric data; Image J V2.0.0 was used to quantify the images; Statistical analysis was performed using GraphPad Prism 7.

For manuscripts utilizing custom algorithms or software that are central to the research but not yet described in published literature, software must be made available to editors and reviewers. We strongly encourage code deposition in a community repository (e.g. GitHub). See the Nature Research guidelines for submitting code & software for further information.

Data

Policy information about availability of data

All manuscripts must include a data availability statement. This statement should provide the following information, where applicable:

- Accession codes, unique identifiers, or web links for publicly available datasets
- A list of figures that have associated raw data
- A description of any restrictions on data availability

The authors declare that the main data supporting the findings of this study are available within the article and its Supplementary Information. Extra data are available from the corresponding author upon reasonable request. The source data underlying Figs. 1-10 and Supplementary Figs. 1-5, 8, 10-17, 19-23, 25-29, 31-33, 35, 37, 40, 42 are provided with the paper as a Source Data file.

Field-specific reporting

Please select the one below that is the best fit for your research. If you are not sure, read the appropriate sections before making your selection.

- [x] Life sciences
- [ ] Behavioural & social sciences
- [ ] Ecological, evolutionary & environmental sciences

For a reference copy of the document with all sections, see nature.com/documents/nr-reporting-summary-flat.pdf

Life sciences study design

All studies must disclose on these points even when the disclosure is negative.

Sample size	The sample sizes of this study were determined on the basis of similar published studies (Liang, et al. Nat Biomed Eng 2019, 3(9): 729-740). In antitumor experiments, 5-6 mice each group were used to analyze tumor volume, 3-6 mice each group were used to analyze tumor weight and microenvironment, and 7-8 mice were used to analyze the survival rate. For SHG and dorsal window chamber imaging, the sample size for each group was 10. For the the co-localization of Cy5-labelled anti-PD-1 antibody and blood-vessel analysis in tumour-bearing mice, the sample size for each group was 15. For other experiments, the sample size for each group was 3-5.
Data exclusions	No data were excluded in the analysis.
Replication	Experiments were repeated and experimental findings were reproducible. Details of experimental replicates are given in the figure legends. All reported attempts at replication were successful.
Randomization	All samples were randomly allocated into experimental groups.
Blinding	Investigators were not blinded to group allocation in tumour volume and weight measurement, because scientists must keep careful track of conditions, it would be exceedingly difficult to blind such studies. However, the treatment efficacy was apparent in both quantification and in representative images of outcomes. Survival data were determined by blinded staff.

Behavioural & social sciences study design

All studies must disclose on these points even when the disclosure is negative.

Study description	*Briefly describe the study type including whether data are quantitative, qualitative, or mixed-methods (e.g. qualitative cross-sectional, quantitative experimental, mixed-methods case study).*
Research sample	*State the research sample (e.g. Harvard university undergraduates, villagers in rural India) and provide relevant demographic information (e.g. age, sex) and indicate whether the sample is representative. Provide a rationale for the study sample chosen. For studies involving existing datasets, please describe the dataset and source.*
Sampling strategy	*Describe the sampling procedure (e.g. random, snowball, stratified, convenience). Describe the statistical methods that were used to predetermine sample size OR if no sample-size calculation was performed, describe how sample sizes were chosen and provide a rationale for why these sample sizes are sufficient. For qualitative data, please indicate whether data saturation was considered, and what criteria were used to decide that no further sampling was needed.*
Data collection	*Provide details about the data collection procedure, including the instruments or devices used to record the data (e.g. pen and paper, computer, eye tracker, video or audio equipment) whether anyone was present besides the participant(s) and the researcher, and whether the researcher was blind to experimental condition and/or the study hypothesis during data collection.*
Timing	*Indicate the start and stop dates of data collection. If there is a gap between collection periods, state the dates for each sample cohort.*
Data exclusions	*If no data were excluded from the analyses, state so OR if data were excluded, provide the exact number of exclusions and the rationale behind them, indicating whether exclusion criteria were pre-established.*
Non-participation	*State how many participants dropped out/declined participation and the reason(s) given OR provide response rate OR state that no participants dropped out/declined participation.*
Randomization	*If participants were not allocated into experimental groups, state so OR describe how participants were allocated to groups, and if allocation was not random, describe how covariates were controlled.*

Ecological, evolutionary & environmental sciences study design

All studies must disclose on these points even when the disclosure is negative.

Study description	*Briefly describe the study. For quantitative data include treatment factors and interactions, design structure (e.g. factorial, nested, hierarchical), nature and number of experimental units and replicates.*
Research sample	*Describe the research sample (e.g. a group of tagged Passer domesticus, all Stenocereus thurberi within Organ Pipe Cactus National Monument), and provide a rationale for the sample choice. When relevant, describe the organism taxa, source, sex, age range and any manipulations. State what population the sample is meant to represent when applicable. For studies involving existing datasets, describe the data and its source.*
Sampling strategy	*Note the sampling procedure. Describe the statistical methods that were used to predetermine sample size OR if no sample-size calculation was performed, describe how sample sizes were chosen and provide a rationale for why these sample sizes are sufficient.*
Data collection	*Describe the data collection procedure, including who recorded the data and how.*
Timing and spatial scale	*Indicate the start and stop dates of data collection, noting the frequency and periodicity of sampling and providing a rationale for these choices. If there is a gap between collection periods, state the dates for each sample cohort. Specify the spatial scale from which the data are taken*
Data exclusions	*If no data were excluded from the analyses, state so OR if data were excluded, describe the exclusions and the rationale behind them, indicating whether exclusion criteria were pre-established.*
Reproducibility	*Describe the measures taken to verify the reproducibility of experimental findings. For each experiment, note whether any attempts to repeat the experiment failed OR state that all attempts to repeat the experiment were successful.*
Randomization	*Describe how samples/organisms/participants were allocated into groups. If allocation was not random, describe how covariates were controlled. If this is not relevant to your study, explain why.*
Blinding	*Describe the extent of blinding used during data acquisition and analysis. If blinding was not possible, describe why OR explain why blinding was not relevant to your study.*

Did the study involve field work? ☐ Yes ☐ No

Field work, collection and transport

Field conditions	*Describe the study conditions for field work, providing relevant parameters (e.g. temperature, rainfall).*
Location	*State the location of the sampling or experiment, providing relevant parameters (e.g. latitude and longitude, elevation, water depth).*
Access & import/export	*Describe the efforts you have made to access habitats and to collect and import/export your samples in a responsible manner and in compliance with local, national and international laws, noting any permits that were obtained (give the name of the issuing authority, the date of issue, and any identifying information).*
Disturbance	*Describe any disturbance caused by the study and how it was minimized.*

Reporting for specific materials, systems and methods

We require information from authors about some types of materials, experimental systems and methods used in many studies. Here, indicate whether each material, system or method listed is relevant to your study. If you are not sure if a list item applies to your research, read the appropriate section before selecting a response.

Materials & experimental systems

n/a	Involved in the study	
☐	☒	Antibodies
☐	☒	Eukaryotic cell lines
☒	☐	Palaeontology and archaeology
☐	☒	Animals and other organisms
☐	☒	Human research participants
☒	☐	Clinical data
☒	☐	Dual use research of concern

Methods

n/a	Involved in the study	
☒	☐	ChIP-seq
☐	☒	Flow cytometry
☒	☐	MRI-based neuroimaging

Antibodies

Antibodies used

Antibodies used included those for:
(1) Antitumor experiments: anti PD-1 antibody (BioXcells, Catalog: BE0146, clone: RMPI1-14, 100 μg per mouse)
(2) Western blot: anti-MMP9 (Proteintech, Catalog: 10375-2-AP, 1/1000 dilution), anti-MMP14 (Abcam, Catalog: ab51074, 1/1000 dilution), anti-beta actin (CST, Catalog: 4970T, 1/1000 dilution)
(3) Immunocytochemistry: anti-CD86 (CST, Catalog: 19589S, 1/200 dilution), anti-CD206 (Servicebio, Catalog: GB13438, 1/200 dilution), anti-CD209 (ABclonal, Catalog: A9649, 1/200 dilution), anti-CD4 (Servicebio, Catalog: GB11064, 1/200 dilution), anti-CD8 (Servicebio, Catalog: GB13429, 1/200 dilution), anti-FxoP3(Servicebio, Catalog: GB13445, 1/200 dilution), anti-Gr1(Servicebio, Catalog: GB11229, 1/200 dilution), anti-CD31 (Biolegend, Catalog: 102405, 1/100 dilution), anti-IgG (Abcam, Catalog: ab37355, 1/200 dilution), Cy3 conjugated Anti-Rabbit IgG (Servicebio, Catolog: GB21303, 1/300 dilution), Cy3-conjugated Anti-Rat IgG (Servicebio, Catalog: GB21302, 1/300 dilution), Cy5 conjugated Anti-rabbit IgG (Servicebio, Catalog: GB27303, 1/300 dilution), FITC conjugated Anti-Rabbit IgG (Servicebio, Catalog: GB22303, 1/300 dilution), FITC conjugated Anti-Rat IgG (Servicebio, Catalog: GB22302, 1/300 dilution)
(4) Flow cytometer:
For flow cytometric staining: 1,000,000 cells in 100 μl volume were used.
anti-mouse CD45 APC (Biolegend, Catalog: 103112, clone: 30-F11, 1/100 dilution),
anti-mouse/human CD11b PerCP/Cyanine5.5 (Biolegend, Catalog: 101227, clone: M1/70, 1/80 dilution),
anti-mouse/human CD11b FITC (Biolegend, Catalog: 101206, clone: M1/70, 1/200 dilution),
anti-mouse F4/80 Brilliant Violet 421 (Biolegend, Catalog: 123137, clone: BM8, 1/100 dilution),
anti-mouse F4/80 PE (Biolegend, Catalog: 123110, clone: BM8, 1/20 dilution),
anti-mouse CD206 APC (Biolegend, Catalog: 141707, clone: C068C2, 1/50 dilution),
anti-mouse CD11c PE/Cyanine7 (Biolegend, Catalog: 117317, clone: N418, 1/80 dilution),
anti-mouse CD3 PE/Cyanine7 (Biolegend, Catalog: 100220, clone: 17A2, 1/100 dilution),
anti-mouse CD3 PerCP/Cyanine5.5 (Biolegend, cat. No 100218, clone 17A2, 1/20 dilution),
anti-mouse CD3 APC (Biolegend, cat. No100236, clone 17A2, 1/50 dilution),
anti-mouse CD4 PerCP/Cyanine5.5 (Biolegend, Catalog: 100539, clone: RM4-5, 1/80 dilution),
anti-mouse CD4 FITC (Biolegend, Catalog: 100406, clone: GK1.5, 1/200 dilutio),
anti-mouse CD8a Brilliant Violet 421 (Biolegend, Catalog: 100737, clone 53-6.7, 1/20 dilution),
anti-mouse CD8a PE (Biolegend, Catalog: 100707, clone 53-6.7, 1/100 dilution),
anti-mouse CD8a PE/Cyanine7 (Biolegend, cat. No 100722, clone 53-6.7, 1/80 dilution),
anti-mouse CD69 APC (Biolegend, Catalog: 104513, clone H1.2F3, 1/20 diluiton),
anti-mouse IFN-γ APC (Biolegend, Catalog: 505809, clone XMG1.2, 1/20 dilution),
anti-mouse Ly-6G/Ly-6C (Gr-1) Brilliant Violet 421 (Biolegend, Catalog: 108433, clone RB6-8C5, 1/20 dilution),
anti-mouse CD25 APC (Biolegend, Catalog: 102011, clone PC61, 1/100 dilution),
anti-mouse FOXP3 Brilliant Violet 421 (Biolegend, Catalog: 126419, clone MF-14, 1/50 dilution),
anti-mouse/human CD44 FITC (Biolegend, Catalog: 103022, clone IM7, 1/20 diluiton),
anti-mouse CD62L PE (Biolegend, Catalog: 104407, colne MEL-14, 1/100 dilution),
anti-human CD14 PE (Biolegend, Catalog: 301805, colne M5E2, 1/20 dilution),
anti-human CD11b APC (Biolegend, Catalog: 301309, colne ICRF44, 1/20 dilution),
anti-human CD80 PE/Cyanine7 (Biolegend, Catalog: 305217, clone 2D10, 1/20 dilution),
anti-human CD206 Brilliant Violet 421 (Biolegend, Catalog: 321125, clone 15-2, 1/20 dilution),
anti-human CD45 FITC (Biolegend, Catalog: 304006, colne HI30, 1/20 dilution),
anti-human CD3 PE/Cyanine7 (Biolegend, Catalog: 344816, colne SK7, 1/20 dilution),
anti-human CD8a PE (Biolegend, Catalog: 300907, colne HIT8a, 1/20 dilution),
anti-human CD69 APC (Biolegend, Catalog: 310909, colne FN50, 1/20 dilution),
anti-human/mouse Granzyme B Recombinant APC (Biolegend, Catalog: 372203, colne QA16A02, 1/20 ditlution),
anti-human IFN-γ APC (Biolegend, Catalog: 506510, colne B27, 1/20 dilution),
which were used according to manufacturers' instructions.

Validation

All antibodies were verified by the manufacturers and each lot has been quality tested. All validation statements can be found on the respective antibody website:
anti-mouse CD45 APC: https://www.biolegend.com/en-gb/products/apc-anti-mouse-cd45-antibody-97,
anti-mouse/human CD11b PerCP/Cyanine5.5: https://www.biolegend.com/en-gb/products/percp-cyanine5-5-anti-mouse-human-cd11b-antibody-4257,
anti-mouse/human CD11b FITC: https://www.biolegend.com/en-gb/products/fitc-anti-mouse-human-cd11b-antibody-347,
anti-mouse F4/80 Brilliant Violet 421: https://www.biolegend.com/en-gb/products/brilliant-violet-421-anti-mouse-f4-80-antibody-7199,
anti-mouse F4/80 PE: https://www.biolegend.com/en-gb/products/pe-anti-mouse-f4-80-antibody-4068,
anti-mouse CD206 APC: https://www.biolegend.com/en-gb/products/apc-anti-mouse-cd206-mmr-antibody-7425,
anti-mouse CD11c PE/Cyanine7: https://www.biolegend.com/en-gb/products/pe-cyanine7-anti-mouse-cd11c-antibody-3086,
anti-mouse CD3 PE/Cyanine7: https://www.biolegend.com/en-gb/products/pe-cyanine7-anti-mouse-cd3-antibody-6060,
anti-mouse CD3 PerCP/Cyanine5.5: https://www.biolegend.com/en-gb/products/percp-cyanine5-5-anti-mouse-cd3-antibody-5596,
anti-mouse CD3 APC: https://www.biolegend.com/en-gb/products/apc-anti-mouse-cd3-antibody-8055,
anti-mouse CD4 PerCP/Cyanine5.5: https://www.biolegend.com/en-gb/products/percp-cyanine5-5-anti-mouse-cd4-antibody-4230,
anti-mouse CD4 FITC: https://www.biolegend.com/en-gb/products/fitc-anti-mouse-cd4-antibody-248,
anti-mouse CD8a Brilliant Violet 421: https://www.biolegend.com/en-gb/products/brilliant-violet-421-anti-mouse-cd8a-antibody-7138,

anti-mouse CD8a PE: https://www.biolegend.com/en-gb/products/pe-anti-mouse-cd8a-antibody-155,
anti-mouse CD8a PE/Cyanine7: https://www.biolegend.com/en-gb/products/pe-cyanine7-anti-mouse-cd8a-antibody-1906,
anti-mouse CD69 APC: https://www.biolegend.com/en-gb/products/apc-anti-mouse-cd69-antibody-3169,
anti-mouse IFN-γ APC: https://www.biolegend.com/en-gb/products/apc-anti-mouse-ifn-gamma-antibody-993,
anti-mouse Ly-6G/Ly-6C (Gr-1) Brilliant Violet 421: https://www.biolegend.com/en-gb/products/brilliant-violet-421-anti-mouse-ly-6g-ly-6c-gr-1-antibody-7201,
anti-mouse CD25 APC: https://www.biolegend.com/en-gb/products/apc-anti-mouse-cd25-antibody-420,
anti-mouse FOXP3 Brilliant Violet 421: https://www.biolegend.com/en-gb/products/brilliant-violet-421-anti-mouse-foxp3-antibody-12143,
anti-mouse/human CD44 FITC: https://www.biolegend.com/en-gb/products/fitc-anti-mouse-human-cd44-antibody-314,
anti-mouse CD62L PE: https://www.biolegend.com/en-gb/products/pe-anti-mouse-cd62l-antibody-386,
anti-human CD14 PE: https://www.biolegend.com/en-gb/products/pe-anti-human-cd14-antibody-796,
anti-human CD11b APC: https://www.biolegend.com/en-gb/products/apc-anti-human-cd11b-antibody-765,
anti-human CD80 PE/Cyanine7: https://www.biolegend.com/en-gb/products/pe-cyanine7-anti-human-cd80-antibody-6174,
anti-human CD206 Brilliant Violet 421 https://www.biolegend.com/en-gb/products/brilliant-violet-421-anti-human-cd206-mmr-antibody-8996,
anti-human CD45 FITC: https://www.biolegend.com/en-gb/products/fitc-anti-human-cd45-antibody-707,
anti-human CD3 PE/Cyanine7: https://www.biolegend.com/en-gb/products/pe-cyanine7-anti-human-cd3-antibody-6934,
anti-human CD8a PE: https://www.biolegend.com/en-gb/products/pe-anti-human-cd8a-antibody-762,
anti-human CD69 APC: https://www.biolegend.com/en-gb/products/apc-anti-human-cd69-antibody-1674,
anti-human/mouse Granzyme B Recombinant APC:https://www.biolegend.com/en-gb/products/apc-anti-human-mouse-granzyme-b-recombinant-antibody-14429,
anti-human IFN-γ APC: https://www.biolegend.com/en-gb/products/apc-anti-human-ifn-gamma-antibody-1533.

Eukaryotic cell lines

Policy information about cell lines

Cell line source(s)

RAW264.7, H22, 4T1, THP-1 and HepG2 cells were purchased from Type Culture Collection of the Chinese Academy of Sciences (Shanghai, China). Murine dendritic cell line DC2.4 (H-2b) was kindly provided by Dr. Haifang Yin (Tianjin Medical University, Tianjin, China), who obtained it from Dr. De Yang (Center for Cancer Research, NIH, USA). Dr. De Yang obtained this cell line from Dr. K. L. Rock (Dana-Farber Cancer Institute, Boston, MA) who established it (J Immunol, 1997, 158: 2723-2730).

Authentication

Each cell line we used was morphologically confirmed according to the information provided by the cell-source center.

Mycoplasma contamination

Cells were tested negative for mycoplasma contamination by using the MycAway-Color one-step mycoplasma detection kit.

Commonly misidentified lines
(See ICLAC register)

No commonly misidentified cell lines were used.

Palaeontology and Archaeology

Specimen provenance

Provide provenance information for specimens and describe permits that were obtained for the work (including the name of the issuing authority, the date of issue, and any identifying information).

Specimen deposition

Indicate where the specimens have been deposited to permit free access by other researchers.

Dating methods

If new dates are provided, describe how they were obtained (e.g. collection, storage, sample pretreatment and measurement), where they were obtained (i.e. lab name), the calibration program and the protocol for quality assurance OR state that no new dates are provided.

☐ Tick this box to confirm that the raw and calibrated dates are available in the paper or in Supplementary Information.

Ethics oversight

Identify the organization(s) that approved or provided guidance on the study protocol, OR state that no ethical approval or guidance was required and explain why not.

Note that full information on the approval of the study protocol must also be provided in the manuscript.

Animals and other organisms

Policy information about studies involving animals; ARRIVE guidelines recommended for reporting animal research

Laboratory animals

BALB/c mice (male and female, 18 ± 2g, six-to eight-weeks old) and C57BL/6 mice were purchased from Beijing Vital River Laboratory Animal Technology Co., Ltd. (Beijing, China). Mice were housed in an animal facility under constant environmental conditions (room temperature, 21±1°C; relative humidity, 40-70% and a 12-h light-dark cycle). All mice had access to food and water ad libitum.

Wild animals

No wild animals were used.

Field-collected samples	No filed-collection was performed.
Ethics oversight	All animal experiments were approved by the Institutional Animal Care and Use Committee at Tongji Medical College, Huazhong University of Science and Technology (Wuhan, China).

Note that full information on the approval of the study protocol must also be provided in the manuscript.

Human research participants

Policy information about studies involving human research participants

Population characteristics	Patient 1: female, 65 years old, primary hepatic carcinoma, interventional therapy for 4 times in the past.<br>Patient 2: female, 35 years old, liver space-occupying lesions, metastatic liver cancer, no past treatment categories.<br>Fresh HCC tissues were obtained from liver cancer patients undergoing routine surgical resection at the Tongji Hospital, Tongji Medical College of Huazhong University of Science and Technology (Wuhan, China). HCC tissues not required for pathological diagnostic procedures were obtained after surgical resection. Blood was obtained from the same liver cancer patients and then collected in EDTA collection tubes. All samples were stored at 4 °C until processed (within 24 hours).
Recruitment	Patients undergoing routine surgical tumor resection were informed of the study and consented by Tongji Hospital staff. Fresh organotypic samples and peripheral blood from the same patients were provided by surgeons, and the obtained samples were cut into thin slices and then randomly grouped, without potential self-selection bias.
Ethics oversight	The human organotypic slice culture experiment and human peripheral blood monocyte-derived macrophages (MDMs) experiment were approved by the Clinical Trial Ethics Committee of Huazhong University of Science and Technology (Wuhan, China).

Note that full information on the approval of the study protocol must also be provided in the manuscript.

Clinical data

Policy information about clinical studies

All manuscripts should comply with the ICMJEguidelines for publication of clinical research and a completedCONSORT checklist must be included with all submissions.

Clinical trial registration	*Provide the trial registration number from ClinicalTrials.gov or an equivalent agency.*
Study protocol	*Note where the full trial protocol can be accessed OR if not available, explain why.*
Data collection	*Describe the settings and locales of data collection, noting the time periods of recruitment and data collection.*
Outcomes	*Describe how you pre-defined primary and secondary outcome measures and how you assessed these measures.*

Dual use research of concern

Policy information about dual use research of concern

Hazards

Could the accidental, deliberate or reckless misuse of agents or technologies generated in the work, or the application of information presented in the manuscript, pose a threat to:

No	Yes	
☐	☐	Public health
☐	☐	National security
☐	☐	Crops and/or livestock
☐	☐	Ecosystems
☐	☐	Any other significant area

Experiments of concern

Does the work involve any of these experiments of concern:

No	Yes	
☐	☐	Demonstrate how to render a vaccine ineffective
☐	☐	Confer resistance to therapeutically useful antibiotics or antiviral agents
☐	☐	Enhance the virulence of a pathogen or render a nonpathogen virulent
☐	☐	Increase transmissibility of a pathogen
☐	☐	Alter the host range of a pathogen
☐	☐	Enable evasion of diagnostic/detection modalities
☐	☐	Enable the weaponization of a biological agent or toxin
☐	☐	Any other potentially harmful combination of experiments and agents

ChIP-seq

Data deposition

☐ Confirm that both raw and final processed data have been deposited in a public database such as GEO.

☐ Confirm that you have deposited or provided access to graph files (e.g. BED files) for the called peaks.

Data access links<br>*May remain private before publication.*	*For "Initial submission" or "Revised version" documents, provide reviewer access links. For your "Final submission" document, provide a link to the deposited data.*
Files in database submission	*Provide a list of all files available in the database submission.*
Genome browser session<br>(e.g. UCSC)	*Provide a link to an anonymized genome browser session for "Initial submission" and "Revised version" documents only, to enable peer review. Write "no longer applicable" for "Final submission" documents.*

Methodology

Replicates	*Describe the experimental replicates, specifying number, type and replicate agreement.*
Sequencing depth	*Describe the sequencing depth for each experiment, providing the total number of reads, uniquely mapped reads, length of reads and whether they were paired- or single-end.*
Antibodies	*Describe the antibodies used for the ChIP-seq experiments; as applicable, provide supplier name, catalog number, clone name, and lot number.*
Peak calling parameters	*Specify the command line program and parameters used for read mapping and peak calling, including the ChIP, control and index files used.*
Data quality	*Describe the methods used to ensure data quality in full detail, including how many peaks are at FDR 5% and above 5-fold enrichment.*
Software	*Describe the software used to collect and analyze the ChIP-seq data. For custom code that has been deposited into a community repository, provide accession details.*

Flow Cytometry

Plots

Confirm that:

☒ The axis labels state the marker and fluorochrome used (e.g. CD4-FITC).

☒ The axis scales are clearly visible. Include numbers along axes only for bottom left plot of group (a 'group' is an analysis of identical markers).

☒ All plots are contour plots with outliers or pseudocolor plots.

☒ A numerical value for number of cells or percentage (with statistics) is provided.

Methodology

Sample preparation	(1) Cultured cells were trypsinized, washed with PBS for three time, then cells were collected for cytometric analysis;<br>(2) Mouse: For the determination of intracellular accumulation in tumour cells and immune cells and the analysis of tumor immune microenvironment, the tissues were cut into small pieces and incubated in RPMI 1640 medium containing 0.8 mg/mL collagenase type I and 5 μg /ml DNase I at 37 °C for 30 min. The single cell suspensions were harvested by washing twice with PBS and filtering twice through a 40 μm cell strainer. Tumor-infiltrating lymphocytes (TILs) were isolated by Ficoll-Paque

	PLUS density gradient media.<br>(3) Human: Human blood was processed to obtain PBMCs by Ficoll gradient. Tumor slices were washed three times with PBS, minced and digested with 0.8 mg/mL collagenase type I and 5 µg/mL DNase I in RPMI 1640 medium at 37 °C for 1 h. The cells were filtered twice using a 40 µm cell strainer and washed twice with PBS.
Instrument	CytoFLEX S or Beckman Coulter FC500.
Software	The software used for collecting and analyzing was Beckman Coulter cytExpert 2.3.0.84 and CXP Cytometer 2.3.
Cell population abundance	No cell sorting was performed.
Gating strategy	Cells were gated by FSC/SSC gates and then FSC/FSC-width to select single cells. After that the detail gating strategy was showed as follows:<br>Mouse macrophages: CD11b+, F4/80+,<br>Mouse DCs: CD45+, F4/80-, CD11c+,<br>Mouse M1-like Tumor associated macrophages (TAMs): CD11b+, F4/80+, CD11c+,<br>Mouse M2-like TAMs: CD11b+, F4/80+, CD206+,<br>Mouse CD4+ T cells: CD45+, CD3+, CD4+ (Expression of CD69 was examined),<br>Mouse CD8+ T cells: CD45+, CD3+, CD8a+ (Expression of CD69, IFN-γ was examined),<br>Mouse myeloid-derived suppressor cells (MDSCs): CD45+, CD11b+, Gr1+,<br>Mouse regulatory T cells (Tregs): CD45+, CD4+, CD25+, FoxP3+,<br>Mouse effector memory T cells: CD3+, CD8+, CD44+, CD62L-,<br>Human CD8+ T cells: CD45+, CD3+, CD8a+ (Expression of CD69, IFN-γ and Granzyme B was examined),<br>Human M1-like TAMs: CD11b+, CD14+, CD80+,<br>Human M2-like TAMs: CD11b+, CD14+, CD206+.

☒ Tick this box to confirm that a figure exemplifying the gating strategy is provided in the Supplementary Information.

Magnetic resonance imaging

Experimental design

Design type	*Indicate task or resting state; event-related or block design.*
Design specifications	*Specify the number of blocks, trials or experimental units per session and/or subject, and specify the length of each trial or block (if trials are blocked) and interval between trials.*
Behavioral performance measures	*State number and/or type of variables recorded (e.g. correct button press, response time) and what statistics were used to establish that the subjects were performing the task as expected (e.g. mean, range, and/or standard deviation across subjects).*

Acquisition

Imaging type(s)	*Specify: functional, structural, diffusion, perfusion.*
Field strength	*Specify in Tesla*
Sequence & imaging parameters	*Specify the pulse sequence type (gradient echo, spin echo, etc.), imaging type (EPI, spiral, etc.), field of view, matrix size, slice thickness, orientation and TE/TR/flip angle.*
Area of acquisition	*State whether a whole brain scan was used OR define the area of acquisition, describing how the region was determined.*

Diffusion MRI ☐ Used ☐ Not used

Preprocessing

Preprocessing software	*Provide detail on software version and revision number and on specific parameters (model/functions, brain extraction, segmentation, smoothing kernel size, etc.).*
Normalization	*If data were normalized/standardized, describe the approach(es): specify linear or non-linear and define image types used for transformation OR indicate that data were not normalized and explain rationale for lack of normalization.*
Normalization template	*Describe the template used for normalization/transformation, specifying subject space or group standardized space (e.g. original Talairach, MNI305, ICBM152) OR indicate that the data were not normalized.*
Noise and artifact removal	*Describe your procedure(s) for artifact and structured noise removal, specifying motion parameters, tissue signals and physiological signals (heart rate, respiration).*
Volume censoring	*Define your software and/or method and criteria for volume censoring, and state the extent of such censoring.*

Statistical modeling & inference

Model type and settings | *Specify type (mass univariate, multivariate, RSA, predictive, etc.) and describe essential details of the model at the first and second levels (e.g. fixed, random or mixed effects; drift or auto-correlation).*

Effect(s) tested | *Define precise effect in terms of the task or stimulus conditions instead of psychological concepts and indicate whether ANOVA or factorial designs were used.*

Specify type of analysis: ☐ Whole brain ☐ ROI-based ☐ Both

Statistic type for inference (See Eklund et al. 2016) | *Specify voxel-wise or cluster-wise and report all relevant parameters for cluster-wise methods.*

Correction | *Describe the type of correction and how it is obtained for multiple comparisons (e.g. FWE, FDR, permutation or Monte Carlo).*

Models & analysis

n/a	Involved in the study
☐	☐ Functional and/or effective connectivity
☐	☐ Graph analysis
☐	☐ Multivariate modeling or predictive analysis

Functional and/or effective connectivity | *Report the measures of dependence used and the model details (e.g. Pearson correlation, partial correlation, mutual information).*

Graph analysis | *Report the dependent variable and connectivity measure, specifying weighted graph or binarized graph, subject- or group-level, and the global and/or node summaries used (e.g. clustering coefficient, efficiency, etc.).*

Multivariate modeling and predictive analysis | *Specify independent variables, features extraction and dimension reduction, model, training and evaluation metrics.*
